# Rapid Emergence of T Follicular Helper and Germinal Center B Cells Following Antiretroviral Therapy in Advanced HIV Disease

**DOI:** 10.3389/fimmu.2021.752782

**Published:** 2021-12-01

**Authors:** Chun-Shu Wong, Clarisa M. Buckner, Silvia Lucena Lage, Luxin Pei, Felipe L. Assis, Eric W. Dahlstrom, Sarah L. Anzick, Kimmo Virtaneva, Adam Rupert, Jeremy L. Davis, Ting Zhou, Elizabeth Laidlaw, Maura Manion, Frances Galindo, Megan Anderson, Catherine A. Seamon, Michael C. Sneller, Andrea Lisco, Claire Deleage, Stefania Pittaluga, Susan Moir, Irini Sereti

**Affiliations:** ^1^ Laboratory of Immunoregulation, National Institute of Allergy and Infectious Diseases (NIAID), National Institutes of Health (NIH), Bethesda, MD, United States; ^2^ Rocky Mountain Laboratories, National Institute of Allergy and Infectious Diseases (NIAID), National Institutes of Health (NIH), Hamilton, MT, United States; ^3^ Leidos Biomedical Research Inc., Frederick National Laboratory for Cancer Research, Frederick, MD, United States; ^4^ Center for Cancer Research, National Cancer Institute, National Institutes of Health (NIH), Bethesda, MD, United States; ^5^ Laboratory of Pathology, Center for Cancer Research, National Cancer Institute, National Institutes of Health (NIH), Bethesda, MD, United States; ^6^ Intramural Clinical Management and Operations Branch, National Institute of Allergy and Infectious Diseases (NIAID), National Institutes of Health (NIH), Bethesda, MD, United States; ^7^ Critical Care Medicine, Clinical Center, National Institutes of Health (NIH), Bethesda, MD, United States; ^8^ AIDS and Cancer Virus Program, Leidos Biomedical Research, Frederick National Laboratory for Cancer Research, Frederick, MD, United States

**Keywords:** HIV, T follicular helper cells, germinal centers, interferon, reconstitution

## Abstract

Low nadir CD4 T-cell counts in HIV^+^ patients are associated with high morbidity and mortality and lasting immune dysfunction, even after antiretroviral therapy (ART). The early events of immune recovery of T cells and B cells in severely lymphopenic HIV^+^ patients have not been fully characterized. In a cohort of lymphopenic (CD4 T-cell count < 100/µL) HIV^+^ patients, we studied mononuclear cells isolated from peripheral blood (PB) and lymph nodes (LN) pre-ART (n = 40) and 6-8 weeks post-ART (n = 30) with evaluation of cellular immunophenotypes; histology on LN sections; functionality of circulating T follicular helper (cTfh) cells; transcriptional and B-cell receptor profile on unfractionated LN and PB samples; and plasma biomarker measurements. A group of 19 healthy controls (HC, n = 19) was used as a comparator. T-cell and B-cell lymphopenia was present in PB pre-ART in HIV^+^ patients. CD4:CD8 and CD4 T- and B-cell PB subsets partly normalized compared to HC post-ART as viral load decreased. Strikingly in LN, ART led to a rapid decrease in interferon signaling pathways and an increase in Tfh, germinal center and IgD^-^CD27^-^ B cells, consistent with histological findings of post-ART follicular hyperplasia. However, there was evidence of cTfh cells with decreased helper capacity and of limited B-cell receptor diversification post-ART. In conclusion, we found early signs of immune reconstitution, evidenced by a surge in LN germinal center cells, albeit limited in functionality, in HIV^+^ patients who initiate ART late in disease.

## Introduction

Effective antiretroviral therapy (ART) has changed the management of HIV infection from a progressive immune deficiency with life-threatening opportunistic infections to a chronic inflammatory disease (1, 2). Although ART successfully suppresses viral replication, morbidity and mortality remain high the first 6-12 months of therapy in patients who have severe CD4 T-cell lymphopenia before initiating therapy (3–5). Late presentation with CD4 T cells < 200 or even < 100 cells/µL, is not uncommon in most resource limited settings and many Western urban centers, especially in minorities. Restoration of full immunologic function is rarely achieved in patients presenting late for ART initiation (6, 7), who also appear to be at higher risk for non-communicable complications of HIV such as cardiovascular disease, non-AIDS malignancies, frailty and neurocognitive disorders despite virologic suppression (8–10). Although the mechanisms involved in residual immunological dysfunction remain unclear, older age, co-infections, and chronic immune activation linked with low nadir CD4 T-cell counts have been identified as predisposing factors (11–15).

Several studies have focused on the peripheral blood in an attempt to delineate the dynamics of immune cell restoration following initiation of ART. CD4 T-cell reconstitution in the peripheral blood has been characterized by initial increases, that to some extent may represent tissue redistribution and improved survival of memory CD4 T cells, followed by a slower recovery of naïve CD4 T cells (16). With respect to B cells, several of the phenotypic and functional abnormalities of B cells that have been described in the absence of ART are in part due to the immune activating effects of the virus and reverse with ART (17). In advanced HIV disease, immature/transitional B cells are particularly over-represented in the absence of ART and associate with CD4 T-cell lymphopenia (18).

Lymphoid tissues play an important role in the pathogenesis and persistence of HIV infection. Upon encounter with foreign antigens, responding follicles become activated and develop into germinal centers (GC); these are highly dynamic structures where antigen-specific B cells undergo affinity maturation with help from specialized CD4 T cells, known as T follicular helper (Tfh) cells (19). In humans, the processes associated with normal GC development, as well as those perturbed by disease or involved in immune reconstitution, have not been well studied. Nonetheless, chronic inflammation and immune activation in people with HIV (PWH) and chronic viremia, are known to have detrimental effects in lymphoid tissues (20–22). In advanced HIV disease, analyses of lymph nodes (LN) have revealed evidence of progressive fibrosis, depletion of fibroblastic reticulum cells and alterations in CD4 T- and B-cell populations (11, 20, 23, 24). Furthermore, LN abnormalities, including follicular hyperplasia, follicular regression, follicular lysis, or the absence of follicles, are not readily reversed with effective ART, as evidenced by their persistence almost two years post-treatment (25). In addition, poor response to vaccines persists years after initiation of ART, suggesting long-term effects of HIV on B-cell function and/or CD4 T-cell help (26).

Relatively little is known regarding the effect of ART on the reconstitution of T and B cells in lymphoid tissues in advanced HIV disease, especially in the critical period immediately following the initiation of ART. Here, we investigated the dynamics of CD4 T-cell and B-cell changes in peripheral blood (PB) and LN following the initiation of ART and reduction of plasma viremia in patients with advanced HIV disease. We show that as ART reduces viral replication with concomitant decreases in lymphoid and peripheral inflammation, there is a strong expansion of LN GC populations, including both GC B cells (GCBC) and Tfh cells. However, evidence of limited improvement in functionality of Tfh cells and modest expansion of the B-cell repertoire suggests that the immune reconstitution may be limited or incomplete.

## Materials and Methods

### Study Design

This study examined HIV^+^ patients with advanced disease (CD4 T-cell counts, <100 cells/µL) before and 6-8 weeks after ART initiation. Biopsies of palpable axillary or inguinal LN and research phlebotomy were performed at the National Institutes of Health (NIH) Clinical Research Center in Bethesda, MD under protocols approved by the National Institute of Allergy and Infectious Diseases (NIAID) Institutional Review Board (ClinicalTrials.gov identifiers: NCT02147405, NCT00001316, and NCT00001281). All participants provided written informed consent. The study included HIV^+^ participants (**Table 1** and **Supplementary Table 1**): 40 who were ART-naïve and 30 who were on ART for 6-8 weeks (including 23 paired pre- and post-ART longitudinal samples). 19 HIV-uninfected participants served as healthy controls, HC (**Table 1** and **Supplementary Table 2**). A portion of tissue was fixed in 4% formalin, and LN mononuclear cells (LNMC) were isolated and used for phenotypic analyses.

**Table 1:** Clinical characteristics of study participants.

Characteristic	Pre-ART (N = 40)	Post-ART (N = 30)	HC (N = 19)
**Age - years**	37 (22-53)	38 (22-53)	40 (19-57)
**Sex (M/F)**	28/12	20/10	12/7
**Race**			
White/Hispanic - N (%)	17 (42)	8 (27)	11 (58)
Black - N (%)	18 (45)	19 (63)	6 (32)
Asian - N (%)	4 (10)	2 (7)	2 (10)
Other - N (%)	1 (3)	1 (3)	0 (0)
**HIV RNA (copies/mL)^a^**	251,030 (14,523-3,449,744)	118 (39-2486)	NA
**CD4 T-cell count (cells/μL)^b^**	**20 (0-80)^c^**	**105 (20-328)^c^**	700 (322 - 1401)
**CD8 T-cell count (cells/μL)^b^**	**523 (131-1547)^d^**	**782 (274-3254)^c^**	284 (180-593)
**CD19 B-cell count (cells/μL)^b^**	**62 (5-806)^c^**	193 (22 - 1021)	171 (95 - 275)
**ART regimen**			
Efavirenz-based - N (%)	NA	7 (23)	NA
INSTI-based - N (%)	NA	19 (63)	NA
Other - N (%)	NA	4 (13)	NA

**^a^** Geometric mean (range).

**^b^** Median (range).

Bold indicates significant difference compared to healthy controls (HC); **^c^** p < 0.0001, **^d^** p = 0.0025.

INSTI, Integrase strand transfer inhibitor; NA, not applicable or not available.

### Phenotypic Analyses

PB mononuclear cells (PBMC) were isolated from blood by Ficoll-Hypaque density gradient centrifugation and LNMC were isolated by mechanical disruption and filtered with a 70-um cell strainer. T- and B-cell multicolor flow cytometric analyses were performed using the fluorochrome-conjugated monoclonal antibodies listed in **Supplementary Table 3**, panels 1-4. Fluorescence-activated cell sorting (FACS) analyses were performed on a FACS Canto II flow cytometer or LSR Fortessa (BD Biosciences), with data analyses performed using FlowJo software version 10 (TreeStar Inc.).

### High-Dimensional Data Analysis of Flow Cytometry Data

opt-SNE and FlowSOM analyses were performed using OMIQ software platform (https://omiq.ai). opt-SNE is a modified version of t-SNE that enables high quality embeddings in the optimal amount of compute time without having to tune algorithm parameters (27). B-cell opt-SNE analysis was performed on a subgroup (n = 33, 11 per group) of participants from whom there were sufficient cells to achieve equal sampling of 10,000 CD19^+^ cells from each FCS file, with 1000 iterations, a perplexity of 30, and theta of 0.5. The following markers were used to generate the B-cell opt-SNE maps: CD19, CD20, CD38, CD10, CD21, CD27, IgA, IgG, IgM and IgD. Resulting opt-SNE maps were fed into the FlowSOM (28) clustering algorithm, in which a new-self organizing map (SOM) was generated using hierarchical consensus clustering and 15 clusters were identified. Heatmap displaying column-scaled z-scores of mean fluorescent intensity (MFI) for individual FlowSOM clusters was generated using OMIQ platform.

### *In Vitro* cTfh Cell Co-Culture Assay

PBMC were thawed in complete 10% FBS RPMI media (Millipore Sigma) with Benzonase® Nuclease (Millipore Sigma). CD4 T cells were isolated by magnetic bead negative selection with the EasySep CD4 isolation kit (STEMCell Technologies). Cells were stained for L/D-AQUA (Thermo Fisher) and then extracellular staining was done using: CD4-BV605 (clone RPA-T4, BD Bioscience), CD45RO-PE-Cy7 (clone UCHL1, BD Biosciences), CD8-APC (clone RPA-T8, BD), CXCR5-BV421(clone J252D4, Biolegend), and CD3-PE (SK7, Biolegend). Stained cells were sorted on a BD FACSAria™. 50,000 sorted CD45RO^+^CXCR5^+^ or CD45RO^+^CXCR5^-^ cells were then plated in a 96 U bottom plate. B cells from a non-related healthy control donor were isolated by magnetic bead negative selection with the EasySep B cell isolation kit (STEMCell Technologies). 50,000 B cells were added to the corresponding 96 U bottom plate in 10% FBS RPMI media (Millipore Sigma) with antiretroviral drugs were added to the culture (200nM raltegravir, 200nM lamivudine) (NIH AIDS reagent program). After 7 days of co-culturing in a 37°C incubator, B-cell differentiation was determined by flow cytometry and absolute cell numbers were quantified using counting beads (Thermo Fisher). Staining for B-cell differentiation was performed using antibodies listed in **Supplementary Table 3**, panel 5.

### Plasma and Serum Biomarker Analysis

Cryopreserved plasma samples of a subset of study participants (**Supplementary Table 1**) were analyzed for multiple biomarkers. Interferon gamma (IFN-γ), tumor necrosis factor (TNF-α), myeloperoxidase (MPO), IL-6, IL-8, IL-10, IL-18, IL-27, monocyte chemoattractant protein 1 (MCP-1), macrophage inflammatory protein 3 alpha (MIP-3α), and interleukin 6 receptor (IL-6R) were measured using a custom multiplex kit by electrochemiluminescence (Meso Scale Discovery). D-dimer was measured by enzyme-linked fluorescent assay on a VIDAS instrument (bioMerieux). Soluble CD14 (sCD14), CXCL13, CXCL9 (Bio-Techne), and human soluble programmed death 1 (PD-1) (MyBioSource) were measured using enzyme-linked immunosorbent assay kits. Cryopreserved serum samples were analyzed for IgG, IgM, IgA and TGF-β1 using electrochemiluminescence (Meso Scale Discovery). Lastly, soluble CD25 (sCD25) (Bio-Techne) was measured by traditional ELISA methods. All assays were performed according to the manufacturer’s instructions. CMV viral load was detected *via* quantitative real-time PCR amplification of whole blood using DNA hybridization probes specific for the CMV genome in the clinical laboratory at NIH Clinical Center. Detectable CMV viremia was defined as CMV viral load >250 copies/mL.

### Viral Antibody Titers for CMV, Influenza, and VZV

Antibody titers were evaluated using the Luciferase Immunoprecipitation Systems (LIPS) assay (29). Briefly, pairs of pre- and post-ART serum samples were diluted 1:10 in assay buffer A (20 mM Tris, pH 7.5, 150 mM NaCl, 5 mM MgCl_2_, 1% Triton X-100). 10 µL of diluted human serum, 40 µl of buffer A and 50 µL of 1 x 107 light units of Ruc0-antigen Cos1 cell extract were incubated for 1 hour at room temperature. 7 µL of 30% suspension of Ultralink protein A/G beads was added and incubated for 1 hour at room temperature. After 10 washes with buffer A and 2 washes with PBS, LU was measured in a Berthold LB 960 Centro microplate luminometer using coelenterazine substrate mix (Promega).

### RNA-Seq and BCR Sequencing

Longitudinal transcriptional analyses were performed on 12 HIV^+^ patients (**Supplementary Table 1**). Pairs of pre- and post-ART samples were processed and RNA-seq and B-cell receptor (BCR) sequencing performed. Samples were processed in a single batch to minimize technical noise in the final dataset. PB (unfractionated) samples were extracted using the PAXgene 96 Blood RNA Kit (Qiagen) following the manufacturer’s instructions. LN sections stored in RNAlater solution were homogenized in one ml of Trizol (Thermofisher scientific) in a FastPrep Green lysing matrix vial for 30 seconds at 6.5 m/s in FastPrep^®^-24 instrument (MP Biomedicals). Trizol lysate was combined with 200µL of 1-bromo-3-chloropropane (Millipore-Sigma) and centrifuged at 4°C at 16,000 x g for 15 minutes. The RNA aqueous phase was extracted with an RNeasy 96 kit, according to manufacturer’s recommendations that included an additional on-column Dnase I treatment (Qiagen). RNA purity and concentration were determined by spectrophotometry. PB and LN RNA median yield was 6.0µg (range: 0.6-32.6µg) and 19.3µg (1.0-98.3µg), respectively. RNA quality was assessed using 2100 Bioanalyzer RNA Pico 6000 kit (Agilent Technologies). PB and LN RNA integrity number (RIN) was 9.1 (8.4 to 9.9) and 7.0 (5.7 to 8.7), respectively. PB and LN RNA samples were subjected to an additional purification step using Agencourt RNAClean XP beads for LN samples (Beckman Coulter Life Sciences) and PB samples were further purified using the Globin Removal Mix and instructions provided in the TruSeq^®^ Stranded Total RNA Sample Preparation Guide, protocol #15021048, Rev E (Illumina). The TruSeq^®^ Stranded mRNA Sample Preparation Kit was used to prepare sequencing libraries, as specified in the manufacturer’s recommended procedure, using the RNA Adapter Plate for dual-indexing (Illumina, Guide, Part# 15031047, Rev. E). TruSeq^®^ libraries were quantified on the CFX96 Touch real-time PCR instrument (BioRad) using the Kapa Library Quant Universal qPCR mix and kit instructions (Kapa Biosystems). All samples were individually sized and normalized to a 2 nM concentration. Samples were combined in equimolar ratios to create a single pool and sequenced as 2 X 93 bp reads on the HiSeq 2500 instrument using the HiSeq Rapid SBS 200 cycle kit, according to the manufacturer’s recommended procedure (Illumina). The paired end sequence reads were prepared by first removing any adapter sequences with CutAdapt v1.12 (30) then low quality sequences were filtered and trimmed using the FASTX Toolkit (31). Remaining reads were then mapped to the *H. sapiens* genome GRCh38 using HiSat2 v2.0.5 (32) with strict pairing required. Differential expression analysis was performed using DESeq2 (33) with low/no expressing genes removed and the standard median ratio normalization method applied. Analyses of differentially regulated genes with at least a log_2_ change of 1.3 and P < 0.05 were performed using Ingenuity and Path Designer by Qiagen.

For BCR analyses, RNA from the RNAseq preparation was re-purified (Zymo Research) and 5 ug of RNA was added to each cDNA reaction (Thermo Fisher). Amplification, library preparation, sequencing, and preliminary bioinformatics analysis were performed by Adaptive Biotechnologies. In brief, samples were sequenced with the immunoSEQ human *IGHV* assay using deep-level resolution to identify and quantitate BCR *IGHV* sequences (34). The somatically rearranged CDR3 of these loci was amplified from cDNA using a 2-step, amplification bias-controlled multiplex PCR approach (35, 36). CDR3 libraries were sequenced. A suite of custom algorithms has been developed by Adaptive Biotechnologies to verify, align and catalog the CDR3 sequences. To access and remove PCR bias from the multiplex PCR assay, a synthetic immune system with all V-J combinations was precisely quantitated (35). The data were subsequently analyzed using the ImmunoSEQ Analyzer 3.0. Briefly, nearest-neighbor clustering was used to collapse reads into clonotypes which were then corrected computationally for PCR bias to generate the total number of templates in each sample (35). The CDR3 sequences were then annotated using IMGT (37), and numbers of total and unique productive and unproductive rearrangements were counted. Abundancy comparisons of unique rearrangements were performed using normalized data, which was calculated by randomly downsampling each repertoire to the lowest number of clonotypes across the entire data set. The average of 100 independent computational downsamplings was reported. Comparisons of the BCR repertoire were performed using Simpson clonality, a method of quantifying the unevenness of the frequencies of immune clones present in a repertoire, yielding values between 0 and 1; and Morisita overlap analysis, a measurement of similarity between two datasets, yielding values between 0 and 1 (38).

### Histology

Each specimen was fixed in 10% formalin and routinely processed into paraffin. Hematoxylin and eosin (H&E)-stained sections, as well as pertinent immunohistochemical staining and special staining when available, from a subgroup of participants (**Supplementary Table 4**) were evaluated in a blinded fashion by two hematopathologists. Images were taken with an Olympus Bx50 microscope, Olympus Plan 10X/0.25, 20X/0.40 ∞/0.17, 40X/0.65 and 100X/1.25 ∞/- oil with an adaptor U-TV0.5xC using a Nikon DS-Fi3 camera, using NIS-elements imported into Adobe Photoshop CC 2019. Representative images for various histologic findings were selected prior to group unblinding.

### Immunohistochemistry and Image Analysis

Immunohistochemistry (IHC) and quantitative image analysis were performed on 5-μm tissue sections mounted on glass slides, as previously described in samples from a total of 15 study participants (39). In brief, IHC was performed using a biotin-free polymer approach (Golden Bridge International) on 5-μm tissue sections mounted on glass slides, which were dewaxed and rehydrated with double-distilled water. Multistaining of CD4/CD68/CD163 to quantify CD4^+^ T cells was performed. All slides were scanned at high magnification (×200) using the ScanScope AT2 System (Aperio Technologies), yielding high-resolution data from the entire tissue section. All B-cell follicles were selected (pen tool drawing on 2 tissue sections) and high-resolution images were extracted from these whole-tissue scans. The percent of each defined area occupied by the chromogen (CD4 or CD20 target) was calculated using CellProfiler v3.1.5.

### Statistical Analysis

Statistical analyses were performed using GraphPad Prism (GraphPad Software). For comparisons of variables between groups that contained both paired and unpaired data, a bootstrapped Welch Two Sample t-test (10,000 iterations) was performed using the boot.t.test function of R package MKinfer (40, 41) and the Wilcoxon signed rank test was used for comparisons of paired pre- and post-ART variables where the Friedman global test was first performed. The Spearman’s rank test was used to assess correlations and the Fisher’s exact test was used in the histology grouping data analysis. P values of less than 0.05 were considered significant and adjustments for multiple testing were performed as previously described (22).

## Results

### Increased B- and T-Cell Counts and Hemoglobin Levels Post-ART

To investigate changes in T- and B-cell populations in advanced HIV disease after initiation of ART, we studied three groups of participants (**Table 1**): 40 HIV^+^ patients with a CD4 T-cell count < 100 cells/µL who had not been receiving ART, 30 HIV^+^ patients on ART for 6-8 weeks (all with nadir CD4 < 100 cells/µL) and 19 HIV^-^ individuals, referred to as healthy controls (HC). Of the 40 HIV^+^ ART-naïve participants, 23 were studied both before starting ART and after the initiation of ART, representing a longitudinal subgroup. PB and LN samples were obtained at baseline (before the initiation of ART) and 6-8 weeks after initiation of ART. There were no differences in age and sex between the groups (**Table 1**). Compared to HC, CD4 T-cell counts were lower while CD8 T-cell counts were higher in HIV^+^ patients both pre- and post-ART (**Table 1**). B-cell counts were lower in the HIV^+^ patients before initiation of ART compared to HC. Notably, most of the HIV^+^ participants had opportunistic infections and other co-infections (**Supplementary Table 1**), and most also received an integrase strand transfer inhibitor (INSTI)-based regimen (**Table 1**). Significant changes were observed following the initiation of ART: HIV plasma viremia decreased while CD4 and CD8 T cells, and B-cells increased (**Supplementary Figures 1A–E**). Taken together, the reduction in viremia by ART led to changes in all three lymphocyte populations, although CD4 T-cell counts remained lower than those of HC.

### Dysfunctional Tfh Cells Are the Major Reconstituting CD4 T-Cell Subset After ART

**Figure 1:**
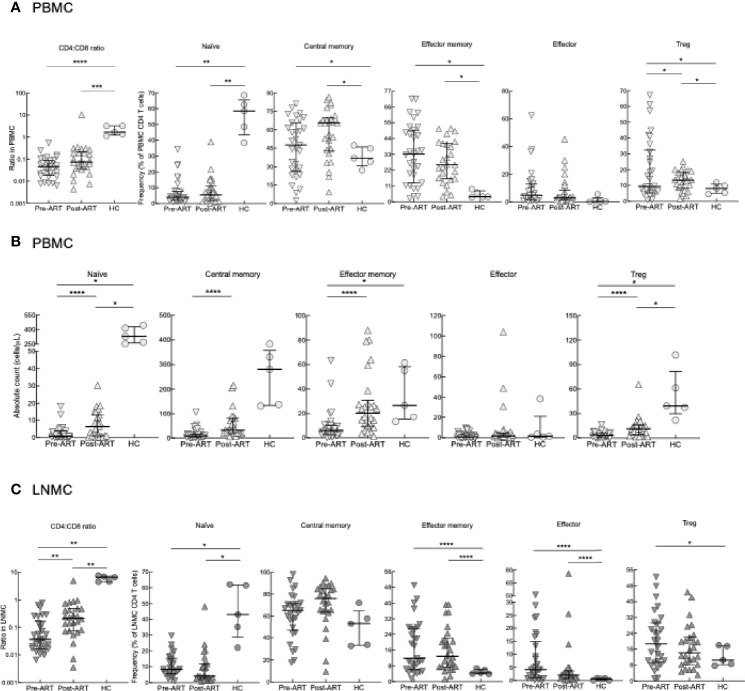
CD4 T-cell subset phenotyping. **(A)** Frequencies of CD4 T-cell subsets in PBMC of pre-ART HIV^+^ (n = 31), 6-8 weeks post-ART (n = 26) and HC (n = 5) participants. **(B)** Absolute counts of T-cell subsets in PBMC of HIV^+^ (n = 31), 6-8 weeks post-ART (n = 26) and HC (n = 5) participants. **(C)** Frequencies of CD4 T-cell subsets in LNMC of pre-ART (n = 29), post-ART (n = 26), or HC (n = 5) participants. *p < 0.05, **p < 0.01, ***p < 0.001, ****p < 0.0001 by bootstrapped Welch Two Sample t-test with 10,000 iterations on full set; data are not statistically significant unless noted.

To investigate the dynamics of T-cell reconstitution early after ART in advanced HIV disease, we performed extensive immunophenotyping of CD4 T cells among PBMC and LN LNMC pre- and post-ART. We utilized markers to identify CD4 T-cell differentiation status: naïve (CD27^+^CD45RO^-^), central memory (CD27^+^CD45RO^+^), effector memory (CD27^-^CD45RO^+^), effector (CD27^-^CD45RO^-^) and regulatory T (Treg; FOXP3^+^CD25^+^) cells (**Figure 1A**). At pre-ART, the CD4:CD8 ratio in PBMC was lower compared to HC and while the ratio did rise post-ART, it remained lower when compared to HC (**Figure 1A**). Among the CD4 T cells, frequencies of naïve, central memory, effector memory, and effector subsets did not differ significantly between pre- and post-ART timepoints, with only Treg CD4 T cells increasing significantly post- compared to pre-ART. When compared to HC, HIV^+^ patients at pre- and post-ART had lower frequencies of naïve but higher frequencies of effector memory, central memory and Treg CD4 T-cell subsets and these differences did not normalize after ART (**Figure 1A**). When comparing absolute counts of CD4 T cells, however, all subsets except effector cells were increased post-ART compared to pre-ART but remained lower when compared to HC except for effector cells (**Figure 1B**).

Similar to PBMC, CD4:CD8 ratios in LNMC were lowest in HIV^+^ patients pre-ART and increased post-ART but did not normalize when compared to HC (**Figure 1C**). Frequencies of LNMC naïve, effector memory, central memory, effector, and Treg CD4 T cells remained largely unchanged pre-versus post-ART. Frequencies of LNMC naïve CD4 T cells were higher in HC compared to HIV^+^ patients both pre- and post-ART, while those of effector memory and effector CD4 T cells were lower (**Figure 1C**). In accordance to PBMCs, Treg CD4 T-cell frequencies were higher pre-ART compared to HC.

**Figure 2:**
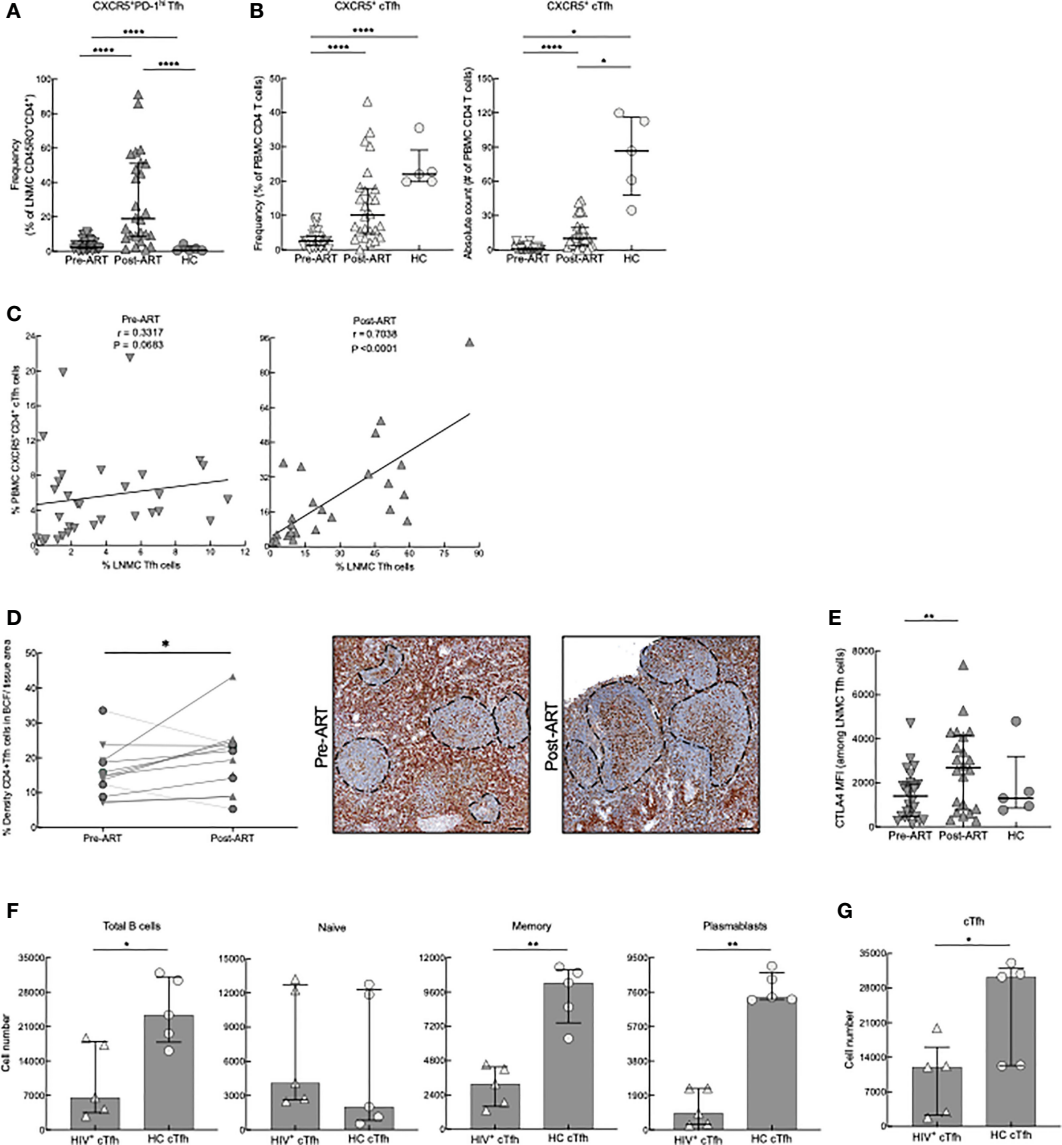
Tfh- and cTfh cell phenotyping and *in vitro* function. **(A)** Frequencies of CXCR5^+^PD-1^hi^ Tfh cells of pre-ART (n = 33), post-ART (n = 26) and HC (n = 5) participants. ****p < 0.0001 by bootstrapped Welch Two Sample t-test with 10,000 iterations on full set; data are not statistically significant unless noted. **(B)** Frequencies and absolute counts of CXCR5^+^ CD45RO^+^ cTfh cells pre-ART (n = 33), post-ART (n = 26), and HC (n = 5) participants. *p <0.05, ****p < 0.0001 by bootstrapped Welch Two Sample t-test with 10,000 iterations on full set; data are not statistically significant unless noted. **(C)** Correlation between pre-ART PBMC cTfh and pre-ART LNMC Tfh cells (left panel) and between post-ART PBMC cTfh versus post-ART LNMC Tfh cells (right panel). Spearman’s rank correlation; data are not significant unless noted. **(D)** Quantification of Tfh cells *in situ* performed pre-ART (n = 11) and post-ART (n = 12) using double IHC staining for CD4 (brown) and myeloid cells (red) to determine density of CD4^+^ T cells within B-cell follicles (BCF); IRIS indicated by circles. Representative images are shown pre- and post-ART for one patient showing increase of CD4^+^ T cells within BCF. *p < 0.05 by one sample t test and Wilcoxon test. Scale bars are 200um. **(E)** CTLA4 expression on LNMC CXCR5^+^PD-1^hi^ Tfh cells pre-ART (n = 26), post-ART (n = 22), and HC (n = 5) participants. **p < 0.01 by bootstrapped Welch Two Sample t-test with 10,000 iterations on full set; data are not significant unless noted. **(F)** cTfh cells from HC (n = 5) or post-ART HIV^+^ (n = 5) participants cocultured with unrelated HC CD19^+^ B cells. Absolute cell numbers of total B cells and B-cell subsets after 7 days in coculture. **(G)** Absolute numbers of cTfh cells at day 7. *p < 0.05, **p < 0.01 by Mann-Whitney U test.

To further evaluate CD4 T-cell differentiation pre- and post-ART, we identified LNMC Tfh cells by their high expression of PD-1 and CXCR5 (representative plots in **Supplementary Figure 2B**). Frequencies of LNMC Tfh cells in HIV^+^ patients both pre- and post-ART were higher when compared to HC; however, there was also a substantial increase in the frequency of LNMC Tfh cells post-ART when compared to pre-ART (**Figure 2A**). Notably, HIV^+^ patients who were diagnosed with immune reconstitution syndrome (IRIS) shortly after ART had lower frequencies of Tfh cells than those without IRIS (**Supplementary Figures 3A, B**). A similar difference was observed for LNMC central memory, although the effect was opposite for effector CD4 T cells (**Supplementary Figure 3B**). To determine whether the emergence of LNMC Tfh cells in HIV^+^ patients was the result of redistribution from the periphery into the LN, we examined the frequency of circulatory CXCR5^+^ CD4^+^ Tfh (cTfh) cells (**Figure 2B**), as previously defined (42), pre- and post-ART. In contrast to LNMC, cTfh-cell frequencies within PBMC were lower in HIV pre-ART compared to the HC, and while frequencies increased post-ART, they remained lower compared to HC (**Figure 2B**). Frequencies of PBMC cTfh cells correlated with LNMC Tfh cells at post- but not pre-ART (**Figure 2C**), suggesting similar dynamics between the two compartments as opposed to redistribution following ART. Finally, we confirmed this observation *in situ* by assessing the frequency of Tfh within tissue sections and observed an increase post-ART in 9 out of 11 HIV^+^ patients with longitudinal sample (P=0.0356, **Figure 2D**).

To further characterize LNMC Tfh cells in our study participants, we evaluated the expression of CTLA4, a coinhibitory repressor that is critical for optimal B cell help. Deletion of CTLA4 leads to enhanced Tfh cell numbers and function (43–45), while increased expression reduces generation of GC B cells (46). Expression of CTLA4 on Tfh cells was higher in HIV^+^ patients post-ART when compared to pre-ART (**Figure 2E**). In addition, we further phenotyped HC and post-ART cTfhs by assessing expression of CXCR3 and CCR6 (representative plots in **Supplementary Figure 2D**). These markers can be used to subdivide cTfh cells into CXCR3^+^CCR6^−^ cells (Tfh1), CXCR3^−^CCR6^−^ cells (Tfh2), CXCR3^−^CCR6^+^ (Tfh17), and CXCR3^+^CCR6^+^ (Tfh1/17); each subset of cTfh cells has a distinctive helper capability (42). The newly reconstituted cTfh cells from HIV^+^ patients had increased differentiation of cTfh1 cells with a decrease of cTfh17 cells compared to HC (**Supplementary Figure 2D**). To evaluate whether this post-ART increase of cTfh1 subset influenced cTfh helper aptitude, we used an *in vitro* cTfh culturing system. Accordingly, CXCR5^+^ and CXCR5^-^ memory CD4 T cells were sorted from PBMC of HC and HIV^+^ post-ART participants and co-cultured for 7 days (in media containing ART) with CD19^+^ B cells isolated from an unrelated HC. CD4 T-cell help was assessed by evaluating changes in absolute cell numbers of naïve and memory B cells (MBC) and plasmablasts or plasma cells, collectively referred to as antibody-secreting cells (ASC), when cTfh (CD4^+^CD45RO^+^CXCR5^+^) cells were added. When co-cultured with cTfh cells isolated from HIV^+^ participants, total B-cell numbers for all subsets, except for naïve B cells (representative gating strategy in **Supplementary Figure 4A**), were lower compared to co-culturing with HC cTfh cells (**Figure 2F**). By day 7 of coculture, cTfh cell numbers were lower in cultures from HIV^+^ compared to HC participants (**Figure 2G**). Furthermore, when co-cultures were performed with HC and HIV^+^ post-ART participant non-cTfh (CD4^+^CD45RO^+^CXCR5^-^) instead of cTfh cells, there were no differences in B-cell percentages and numbers or CD4^+^CXCR5^-^ cell survival (**Supplementary Figures 4B, C**), suggesting a cTfh specific defect. To further examine this decrease of HIV^+^ cTfh cells, we compared apoptosis and proliferation frequencies *via* Annexin V and Ki-67 respectively of the cTfhs on day 3 of co-culture. HIV^+^ cTfh cells underwent much higher rates of apoptosis when compared to HC, while having much lower frequencies of Ki-67^+^ cTfh cells (**Supplementary Figure 4D**). In contrast, *ex vivo* staining for Ki-67 revealed that HIV^+^ cTfh cells had a significantly higher rate of turnover than HC (**Supplementary Figure 4E**). Taken together, these data show that changes in the CD4 T-cell compartment following ART in advanced HIV disease are characterized by a rapid surge of both LN Tfh cells and cTfh counterparts, which appear suboptimal at providing B-cell help.

### GCBC Are the Major Reconstituting B-Cell Subset After ART

**Figure 3:**
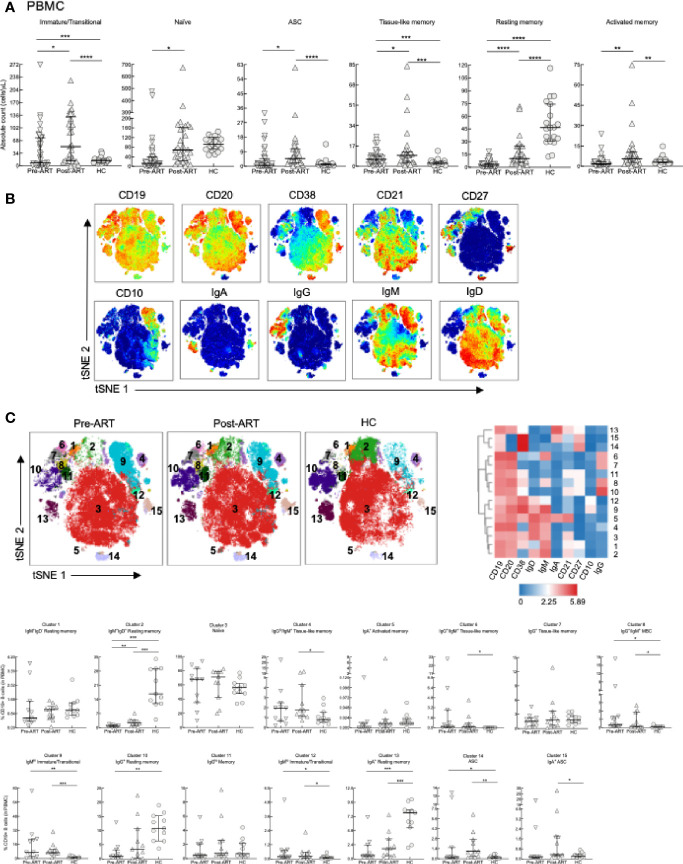
B-cell subset phenotyping in PBMC. **(A)** Absolute counts of B-cell subsets in PBMC of HIV^+^ (n = 39), 6-8 weeks post-ART (n = 29) and HC (n = 19) participants. **(B)** Opt-SNE projections of expression of the indicated markers on CD19^+^ B cells pooled from a subset of participants, pre-ART (n = 11), post-ART (n = 11), and HC (n = 11). **(C)** Opt-SNE projection of pooled CD19^+^ B-cell clusters identified by FlowSOM clustering. Fifteen clusters from FlowSOM analysis were visualized as a heatmap of mean fluorescence intensity, each row as a different cluster, while columns represent analyzed markers, and frequency of B cells from each group in FlowSOM clusters indicated. *p < 0.05, **p < 0.01, ***p < 0.001, ****p < 0.0001 by bootstrapped Welch Two Sample t-test with 10,000 iterations on full set; data are not statistically significant unless noted.

Given that HIV infection has been associated with a number of changes in the B-cell compartment (47), we pursued the investigation of lymphocyte reconstitution following ART by immunophenotyping B cells among PBMC and LNMC of our study participants. PBMC were stained with markers to identify the following B-cell subsets in HIV^+^ patients and HC (**Supplementary Figure 5A**): immature/transitional (CD21^lo/hi^CD10^+^), naïve (CD21^+^CD27^-^), resting MBC (CD21^+^CD27^+^), activated MBC (CD21^lo^CD27^+^), tissue-like MBC (TLM; CD21^lo^CD27^-^), and ASC (CD21^-^CD27^hi^) (48, 49). In HIV^+^ patients, absolute B-cell counts for all subsets were higher post-ART compared to pre-ART (**Figure 3A**). Similar to previous findings (18), absolute counts of immature/transitional B cells were increased compared to HC in both HIV pre- and post-ART (**Figure 3A**). Among mature subsets, absolute counts in HC were higher compared to the two HIV groups for resting MBC (**Figure 3A**). In contrast, and consistent with HIV-associated immune activation, absolute counts were higher in one or both HIV^+^ groups for tissue-like and activated MBC and ASC when compared to HC (**Figure 3A**).

B-cell subsets are often defined by the expression of unswitched (IgM/D) or switched (IgG/A) BCR (50). However, the binding of immunoglobulins to B cells, observed in disease settings such as HIV disease (51), can complicate analyses. Unbiased clustering analyses of high dimensional flow cytometric data can help mitigate this problem and provide further insight of the phenotypic landscape of B cells in various disease settings. Accordingly, global high dimensional mapping with optimized t-distributed stochastic neighbor embedding (opt-SNE) was performed on a subgroup of participants (see details in Materials and Methods) and projected with all samples analyzed for 10 major B-cell markers (**Figure 3B**). FlowSOM analyses identified 15 distinct clusters that were projected by group (**Figure 3C**, top left), delineated by marker intensity with a heat map (**Figure 3C**, top right), and differentiated between groups (**Figure 3C**, bottom). As expected, the largest cluster 3, identified as naïve B cells by expression of IgD/M and CD38 in the absence of CD27 did not differ between groups. In contrast, cell frequencies in clusters 8, representing MBC positive for incompatible isotypes (IgM and IgG), immature/transitional clusters 9 and 12, and ASC cluster 14, were increased pre- and post-ART compared to HC (**Figure 3C**, bottom). Cell frequencies of two TLM clusters 4 and 6, one IgG^lo^/IgM^+^ and the other IgG^+^/IgM^+^, and ASC cluster 15, were increased post-ART compared to HC (**Figure 3C**, bottom). Finally, clusters 2, 10 and 13, which represent IgM^+^/D^+^, IgG^+^ and IgA^+^ resting (CD21^+^CD27^+^) MBC, respectively, had cell frequencies that were higher in HC compared to pre- or post-ART or both (**Figure 3C**, bottom).

**Figure 4:**
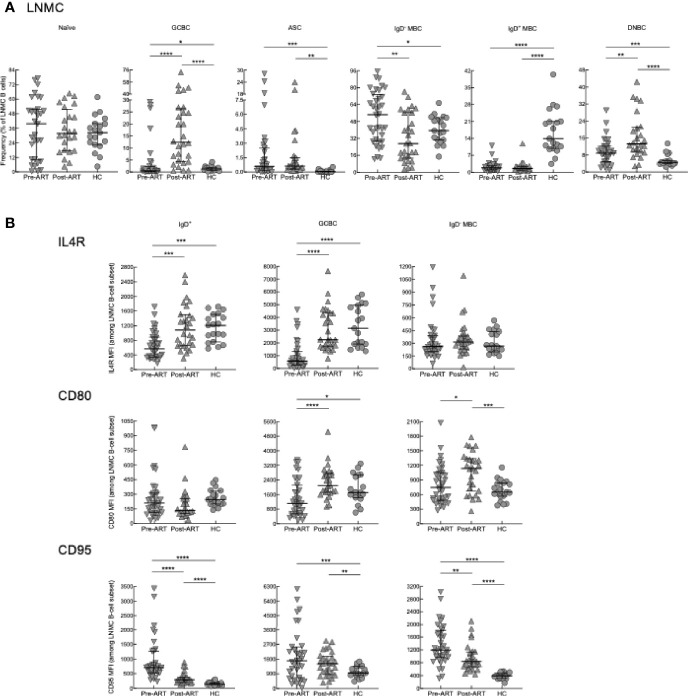
B-cell subset phenotyping and surface marker expression in LN. **(A)** Frequencies of B-cell subsets in LNMC of pre-ART (n = 36), post-ART (n = 28), and HC (n = 19) participants. **(B)** Mean fluorescence intensity (MFI) evaluated for IL4R, CD80 and CD95 on LNMC B-cell subsets of pre-ART (n = 36), post-ART (n = 28), and HC (n = 19) participants. *p < 0.05, **p < 0.01, ***p < 0.001, ****p < 0.0001 by bootstrapped Welch Two Sample t-test with 10,000 iterations on full set; data are not statistically significant unless noted.

In LNMC, B-cell subsets were identified as previously described using CD38, CD27, and IgD (**Supplementary Figure 5B**) (22, 26). There were no significant differences in naïve B cells (IgD^+^CD27^-^) between the three groups (**Figure 4A**). Contrary to expectations based on previous findings (22), yet consistent with Tfh-cell dynamics reported here, frequencies of GCBC (IgD^-^CD38^+^) in HIV pre-ART were similar to those of HC but increased substantially post-ART compared to pre-ART and HC (**Figure 4A**). An approximation of absolute B-cell counts performed by quantitative imaging of LN tissue sections stained with anti-CD20 did not find significant differences in abundance of B cells between pre- and post-ART **(** **Supplementary Figures 5C, D**). This suggests that the increased frequency of GCBC post-ART was not due to a difference in total B cells and could even be an underestimate given that the intensity of CD20 is substantially lower on GCBC than other B cells (22). Frequencies of IgD^-^ MBC (IgD^-^CD27^+^) in HIV pre-ART were also similar to those of HC and higher compared to HIV post-ART (**Figure 4A**). For IgD^+^ MBC (IgD^+^CD27^+^), frequencies were lower in HIV^+^ patients both pre-ART and post-ART compared to HC, whereas for ASC (IgD^-^CD38^hi^), frequencies were higher in HIV^+^ patients both pre-ART and post-ART compared to HC (**Figure 4A**). Lastly, frequencies of a population of IgD^-^CD27^-^ or double-negative B cells (DNBC), that has recently been characterized in LN of patients with COVID-19 (52), was lower in HC compared to both HIV groups, and increased post-ART compared to pre-ART, following similar dynamics as the GCBC (**Figure 4A**). In contrast to T cells, IRIS had no effect on frequencies of LNMC B cells (**Supplementary Figures 6A, B**).

To further characterize LNMC B cells, we performed additional phenotyping of markers which are important for B-T cell interactions and selection of GCBC (53), including the receptor for IL4 (IL4R) and markers of activation CD80 and CD95. For this analysis, IgD^+^ B cells which contain both naïve and IgD^+^ MBC (**Supplementary Figure 5B**), were analyzed as one population. In HIV^+^ pre-ART, intensities of IL4R were lower compared to both HC and HIV^+^ post-ART for IgD^+^ B cells and GCBC but not IgD^-^ MBC (**Figure 4B**). For CD80, intensities were also lower in HIV^+^ pre-ART on GCBC compared to the other two groups, although differences were more nuanced for the two other subsets (**Figure 4B**). For CD95, intensities were higher in HIV^+^ pre- and post-ART compared to HC for all subsets (**Figure 4B**). Among the two HIV groups, CD95 intensities were higher pre- versus post-ART on IgD^+^ B cells and IgD^-^ MBC (**Figure 4B**).

**Figure 5:**
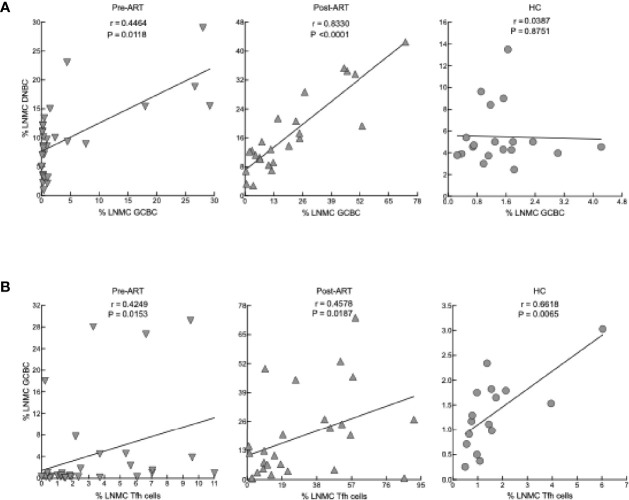
Correlation between cellular phenotypes in LNMC. **(A)** Correlation between frequency of GCBC and DNBC in LNMC of pre-ART (n = 31), post-ART (n = 24) and HC (n = 19) participants. **(B)** Correlation between frequency of Tfh cells and GCBC in LNMC of pre-ART (n = 31), post-ART (n = 25) and HC (n = 16) participants. Spearman’s rank correlation; data are not statistically significant unless noted.

We also considered correlations between the cellular LNMC phenotypes of our participants. Strong direct correlations were observed between frequencies of DNBC and GCBC in HIV^+^ post-ART and to a lesser extent pre-ART but not in HC (**Figure 5A**). Consistent with reporting in chronic HIV viremia (21, 54), there were direct correlations between frequencies of Tfh cells and GCBC in HC and HIV^+^ pre- and post-ART (**Figure 5B**). Taken together, the reduction in viremia by ART during advanced HIV disease led to rapid B-cell changes in LN, characterized by a sharp increase in GCBC and some degree of normalization for markers of activation and interaction with T cells but limited evidence of normalizing subsets in the peripheral blood.

### Rapid Emergence of Follicular Hyperplasia After ART

**Figure 6:**
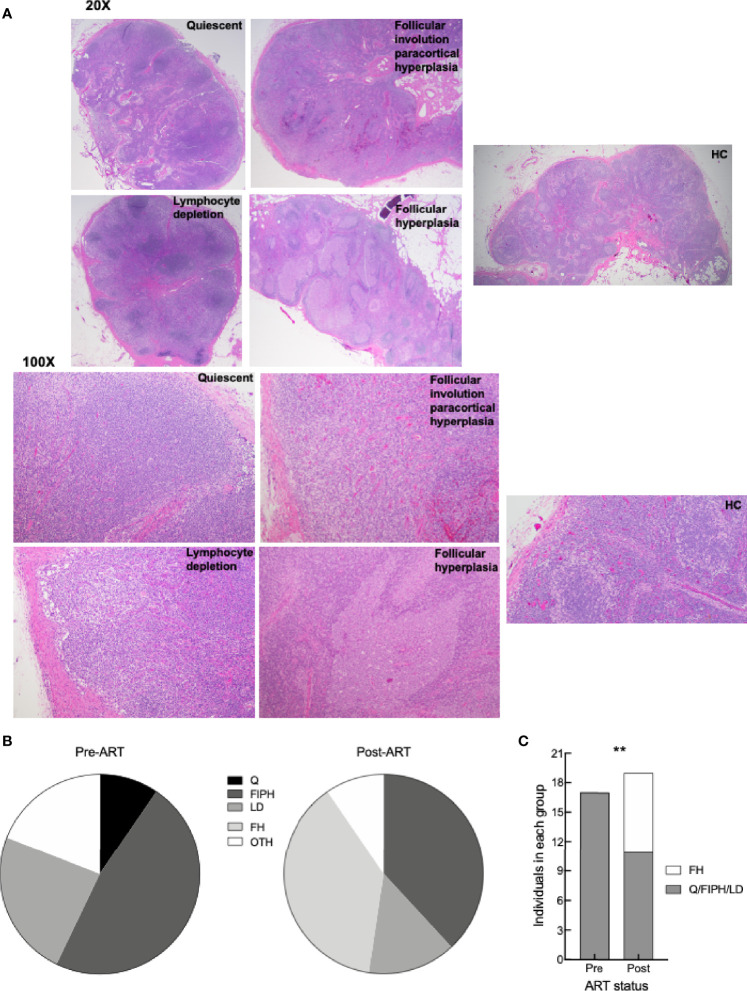
LN histologic characterization. **(A)** Representative images of H&E staining of LN from HC, HIV^+^ pre- and post-ART participants (identifiers in **S1 Table**) depicting the four groups: quiescent (L017 pre-ART), follicular involution with paracortical hyperplasia (L090 pre-ART and HC6), lymphocyte depletion (L061 pre-ART), and follicular hyperplasia (L003 post-ART); 20X and 100X magnification. **(B)** Pie charts depicting proportion of LN pre-ART (n = 21) and post-ART (n = 21) characterized as quiescent (Q), follicular involution with paracortical hyperplasia (FIPH), lymphocyte depletion (LD), follicular hyperplasia (FH), and other (OTH). s**(C)** Proportion of LN pre-ART (n = 17) and post-ART (n = 19) that were grouped as FH or non-FH. **p < 0.01 by Fisher’s exact test.

Elevated frequencies of LN Tfh cells and GCBC in HIV infection during chronic viremia have generally been associated with an expansion of follicular areas and the formation of large often ill-defined GC (24). Given the increased frequencies of Tfh cells and GCBC observed by flow cytometry post-ART in our HIV^+^ cohort, we considered whether these changes were also observed histologically. Consistent with previous observations in HIV disease (55), histologic evaluation performed on HC and paired LN sections of HIV^+^ patients pre- and post-ART revealed four general groups, each based on the predominant finding as illustrated by representative images (**Figure 6A**) and collectively for the 21 pairs (**Figure 6B**), as well as comprehensively for all findings (**Supplementary Table 4**). In addition to the four groups, a number of HIV^+^ patients pre- and/or post-ART were not assigned to a group due to the presence of well-defined co-infections or comorbidities (**Supplementary Table 4**, “other” in **Figure 6B**). Among the four groups, follicular involution with extrafollicular hyperplasia was most frequently observed pre- and post-ART (**Figure 6B**), and typically involved expanded paracortical areas, plasmacytosis and sinus histiocytosis (**Supplementary Table 4**). Another group observed in HIV^+^ pre- and post-ART was lymphocyte depletion, manifested by loss of lymphoid cells in the cortex (**Figure 6A**) and evidence of fibrosis (**Supplementary Table 4**). A third group, quiescent, was observed pre- but not post-ART, and typified by a paucity of secondary follicles (**Figure 6A**). The fourth group, follicular hyperplasia, was absent pre-ART but was observed as the dominant feature post-ART in eight HIV^+^ patients and present in one additional individual in the paracortical hyperplasia group (**Figure 6B** and **Supplementary Table 4**). Furthermore, the presence of follicular hyperplasia relative to the other three groups where secondary follicles were not involved, was enriched post- compared to pre-ART (**Figure 6C**). Thus, while histological findings revealed complex and varied LN features, the presence of follicular hyperplasia was distinctly associated with changes that occurred post-ART and was consistent with phenotypic changes observed post-ART by flow cytometry.

### Transcriptional and BCR Analyses Show Compartmentalization of Immune Reconstitution

**Figure 7:**
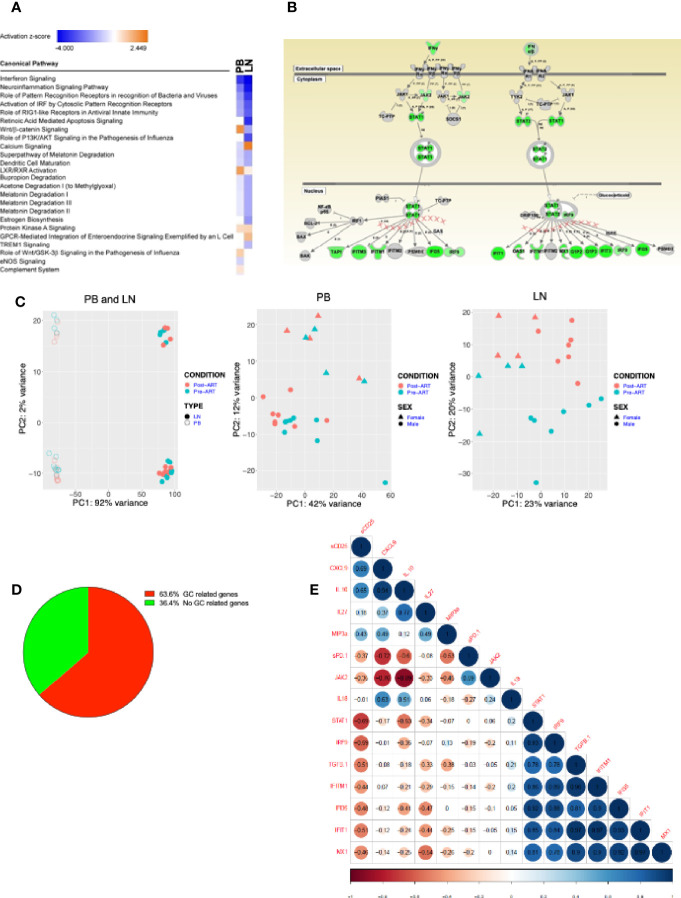
Transcriptional profiling on samples from PB and LN. **(A)** RNAseq of HIV^+^ participant longitudinal samples from PB (n = 12) and LN (n = 11). Differentially expressed genes pre/post ART filtered by a fold change of 1.3, then analyzed by Ingenuity pathway analysis (IPA). Listed are the most significantly enriched pathways. **(B)** Depiction of Type I/II interferon signaling pathway, the most enriched pathway in both PB and LN; green indicates genes significantly downregulated post-ART. **(C)** Principal component analysis (PCA) distinguishing PB (open) and LN (closed) compartments and pre- (green) and post- (red) ART time-points (left panel); PB (middle) and LN (right) panels distinguishing pre- (green) and post- (red) ART time-points and gender: female (triangle) and male (circle). **(D)** Pie chart of LN samples showing percentage of HIV^+^ participant samples with GC-related and non-GC-related genes that underwent a 1.3-fold or more change post-versus pre- ART. **(E)** Correlation matrix of fold changes of plasma biomarkers versus normalized transcripts of interferon related genes (n = 10).

To gain insight into the cellular pathways that are affected pre- and post-ART in our participants with advanced HIV disease, we investigated transcriptional changes in unprocessed cells from the peripheral blood (PB) and LN of a subset of HIV^+^ participants who had LN biopsy both before and after ART initiation (**Supplementary Table 1**). Pathway analysis of differentially expressed genes with at least a log_2_ fold change of 1.3 pre- versus post-ART in the PB and LN revealed several pro-inflammatory genes that were downregulated post-ART, with interferon (IFN) signaling at the top of the list (**Figure 7A**). In further analyses of differentially expressed genes depicted along affected pathways, several downregulated genes were identified in pathways associated with both type I and type II IFN signaling (**Figure 7B**). Furthermore, while enriched pathways were similar in PB and LN, principal component analysis (PCA) nonetheless revealed compartmentalization of gene expression by source of specimen (**Figure 7C**). Of note, clustering by ART status was only observed in LN, suggesting that changes in gene expression changed more rapidly in LN, an observation that was consistent with the immunophenotyping. In addition, by individually comparing patients pre- versus post-ART with a log_2_ fold change of 1.3-fold change or greater, we found genes related to GC were differentially expressed in 64% of patients (**Figure 7D**), confirming the importance of GCBC and Tfh cells in driving LN changes after ART. Cytokines, chemokines and soluble factors that play a role in IFN signaling and HIV pathogenesis were assessed and compared either pre- versus post-ART (**Supplementary Figure 7A**) or as correlations with frequencies of selected LNMC populations (**Supplementary Figure 7B**). Despite the lack of statistically significant differences or correlations for the majority of the 21 biomarkers analyzed, there were notable exceptions: inflammatory biomarkers IL-8, TNFα, MCP-1, and soluble (s) IL6R were elevated in HIV pre-ART when compared to post-ART (**Supplementary Figure 7A**). These extended to direct correlations with LNMC populations pre-ART for TNFα, MCP-1, as well as CXCL13, IgM, sCD14 and sCD25, whereas direct correlations observed both pre- and post-ART were restricted to IgG (**Supplementary Figure 7B**). To determine whether changes in plasma biomarkers could represent surrogates for interferon signaling transcriptomic changes in the LN, we tested for correlations in fold changes of plasma biomarkers to fold changes of normalized transcripts. Of the biomarkers analyzed, TGFβ positively correlated to fold changes of IFIT1, IFI35, MX1, and IFITM1, while sCD25 negatively correlated to STAT1 (**Figure 7E**).

**Figure 8:**
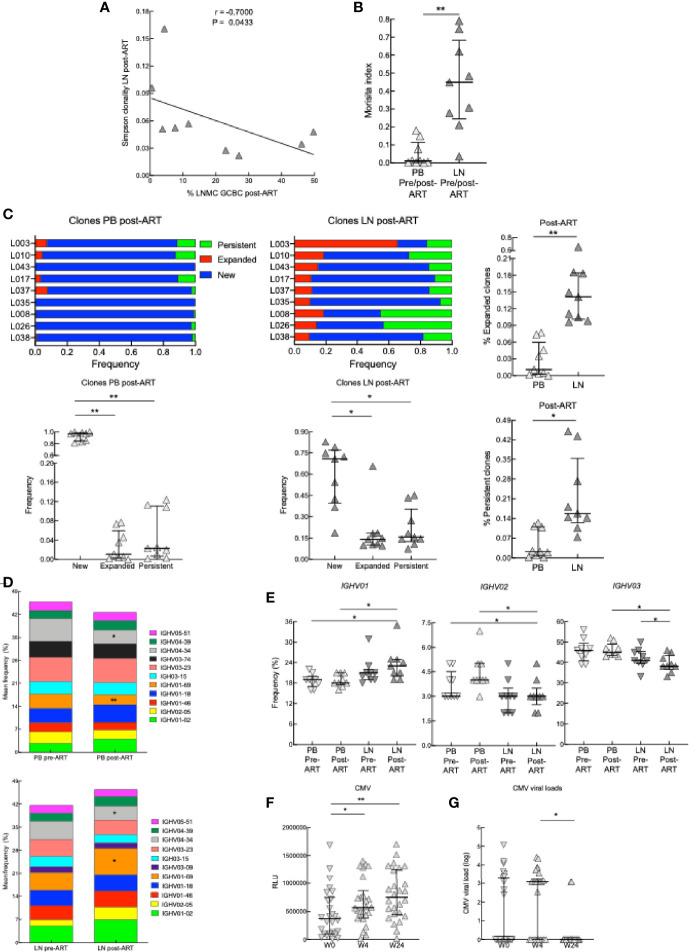
BCR sequencing on samples from PB and LN. **(A)** Correlation between Simpson clonality in LN post-ART and the frequency of GCBC post-ART of nine HIV^+^ pre- and post-ART participants. *p < 0.05 by Spearman’s rank correlation. **(B)** Morisita index determined for PB and LN samples of nine HIV^+^ pre- and post-ART participants. **(C)** Ig heavy chain clone frequencies for nine HIV^+^ participants, depicted and compared at post-ART by categories defined relative to pre-ART: new for clones not previously present, expanded for clones that increased 2-fold and persistent for clones that did not change in frequency. **(D)** Stacked bar graphs showing the average frequency of the top 11 *IGVH* gene families present in PB and LN of nine HIV^+^ pre- and post-ART participants. **(E)** Frequencies of *IGHV01*, *IGHV02* and *IGHV03* families in the PB and LN of nine HIV^+^ pre- and post-ART participants. **(F)** Serum CMV antibody titers at week 0, week 4, or week 24 (n = 26). **(G)** CMV viral loads of 26 HIV^+^ at week 0, 4 or 24. *p < 0.05, **p < 0.01 by Wilcoxon signed rank test after obtaining significance by Friedman test on full set **(C, E)**; data are not statistically significant unless noted.

To further interrogate B cells pre- and post-ART in our HIV^+^ cohort, we sequenced the Ig heavy variable (*IGHV*) genes of PB and LN BCRs in the subset of participants (**Supplementary Table 1**). The total number of unique IgHV sequences was higher in LN than PB both pre- and post-ART (**Supplementary Figure 8A**); however, there were also more total and productive sequences in LN than PB (**Supplementary Table 5**). When downsampling was performed to account for this latter difference between the two compartments, the number of unique *IGHV* sequences was similar between compartments and at both timepoints (**Supplementary Figure 8A**). Furthermore, the analysis of *IGHV* repertoire diversity, as measured by the Simpson clonality index, revealed that while overall clonalities were similar between compartments and at both timepoints (**Supplementary Figure 8B**), the degree of clonality in *IGHV* of LN post-ART correlated inversely with the frequency of GCBC post-ART (**Figure 8A**), suggesting that expanding GCBC associated with a more diverse BCR.

The Morisita index can be used to quantify the degree of similarity in BCR repertoires between two datasets by evaluating the frequencies of clonally related *IGHV* sequences that overlap between datasets (38). When applied to the two compartments analyzed, the degree of similarity pre- versus post-ART was higher in LN than PB (**Figure 8B**), and more clones were retained in LN than PB post-ART (**Supplementary Figure 8C**). In terms of relative abundance of clones over time (pre- versus post-ART), we identified clones that were new or that expanded or persisted within either LN or PB post-ART (**Figure 8C** and **Supplementary Figure 8D**). After ART, while there were more new clones than expanded or persistent clones in both compartments, the latter two types of clones were increased in LN compared to PB (**Figure 8C**). Analysis of *IGHV* family usage in PB and LN revealed that of the top 11 families in PB and LN, 10 were the same pre- and post-ART in each compartment (**Figure 8D**). When compared to pre-ART, family *IGHV01-69* contracted in PB yet expanded in LN while family *IGHV4-34* contracted in both compartments post-ART (**Figure 8D**). Furthermore, when changes in frequencies between compartments and pre-versus post-ART were considered for all members of a family combined, usage of *IGHV01* was increased in LN post-ART relative to PB at both timepoints while usage of *IGHV03* was lower in LN post-ART compared to PB and decreased compared to LN pre-ART (**Figure 8E**). For other families, usage was lower for *IGHV02* in LN post-ART compared to PB at both timepoints while usage of other families across compartments and timepoints were similar (**Figure 8E** and **Supplementary Figure 8E**).

To further investigate the effect of ART on antibody repertoires, we compared IgG titers to common viral co-infections, including CMV, VZV, and influenza. While neither VZV or influenza antibodies increased post-ART (**Supplementary Figure 8F**), those against CMV were increased at 4 weeks post-ART and were maintained at 24 weeks post-ART, despite CMV viral titers having decreased by week 24 (**Figures 8F, G**). In summary, the transcriptional analyses suggest that at post-ART there is decreased type I IFN signaling, increased LN GC signature, changes in the BCR repertoire and diversity between PB and LN compartments, and increased antibody response.

## Discussion

In this study, we examined the dynamics of early immune reconstitution after ART in patients with advanced HIV disease by focusing on CD4 T and B cells that circulate in blood and reside in LN. Here we demonstrate that after initiation of ART, decreased plasma HIV viremia was accompanied by a concomitant rise in B- and T-cell counts. Among PB and LN CD4 T cells, there was an increase in central memory cells after ART as well as a surge of Tfh and cTfh cells. Among LN B cells, there was a similarly rapid and profound expansion of GCBC. These expansions may suggest the beginning of immune reconstitution, especially given the contrasting observation of decreased frequencies of Tfh cells and GCBC in less advanced HIV disease after ART initiation and virologic suppression (54). These changes were accompanied by other evidence of immune reconstitution, including increased CD4:CD8 ratio, decreased type I IFN signaling, evidence of improved ability to mount an antibody response against common pathogens (CMV), and expansion of the BCR repertoire with a shift away from families associated with autoreactivity (*IGHV4-34*) and toward those involved in virus suppression (*IGHV1-69*) (56, 57). The histological findings that follicular hyperplasia in LN only emerged post-ART were also consistent with the immunophenotypic observations on LNMC. However, while collectively the changes that occurred post-ART are potential indicators of immune reconstitution, further work will be needed to determine whether there are direct associations between the changes observed. Furthermore, there were several indicators that the immune reconstitution post-ART was incomplete or inefficient, as evidenced by the i) presence of cTfh cells with poor helper function and survival; ii) B cells with immature and activated profiles in PB and activated/apoptotic profiles in LN; and, iii) a paucity of circulating resting and IgD^+^ MBC in both LN and PB. Whether prolonged ART and sustained virologic suppression will correct and improve the immune reconstitution in PWH who initiate ART in advanced disease remains to be determined.

We found an impressive expansion of Tfh cells early after ART initiation that could not be attributed to redistribution because it was observed in both LN and PB, suggesting that it may be the result of improved homeostatic proliferation and survival. Tfh-cell dynamics have been extensively studied in the context of HIV infection. It has been shown that Tfh-cell numbers increase in HIV infection as CD4 T-cell counts decline, positively correlating with viremia (21, 58). Functionally, this accumulation of Tfh cells is also accompanied by hypergammaglobulinemia and increases in both GCBC and plasma cells (54, 59). However, we did not observe correlations between emerging Tfh cells and related cytokines (TGFβ, IL-27), known to promote their differentiation (19). It is possible that the decrease in type I IFN signaling, a known signaling pathway that skews T helper differentiation towards Th1 rather than Tfh, may have played a role in the emergence of Tfh cells post-ART (60), although there was no correlation between the key genes involved in type I interferon signaling and the frequencies of Tfh cells. Tfh-cell reconstitution was found to negatively correlate with the inflammatory cytokine IL-6, which has been shown to drive Tfh-cell accumulation during chronic SIV infection (61). It is possible that in contrast to the accumulation of Tfh cells during chronic HIV infection where high levels of IL-6 are linked to plasma viremia (61), the reconstitution of a GC-centric phenotype shortly after ART is linked instead with an anti-inflammatory gene signature and decreased proinflammatory cytokines (IL-6, TNFα, MCP-1, IL-6R), as well as decreased type I IFN signaling.

In other conditions of hematopoietic cell recovery such as kidney or bone marrow transplantation, low B-cell counts are associated with slow B-cell reconstitution (62, 63). In contrast, in our cohort, low B-cell counts pre-ART were rapidly normalized post-ART, suggesting a different mechanism of B-cell reconstitution than that reported for transplant recipients. Moreover, it is well known that B-cell subsets in the peripheral blood are altered during HIV disease and only fully normalize when ART is initiated early after infection (48, 49, 64). In advanced HIV disease, frequencies of immature/transitional B cells are particularly elevated, consistent with effects of severe lymphopenia and ongoing viral replication (18). In this study, we found that in addition to increased absolute cell counts of immature/transitional B cells pre-ART, there was a profound depletion of several MBC subsets, particularly IgD^+^ MBC in LN and resting MBC in PBMC. The latter was confirmed by high-dimensional flow cytometry analysis where frequencies of cells in clusters corresponding to IgM/D^+^, IgG^+^ and IgA^+^ resting MBC. These alterations were minimally reversed during the early period following initiation of ART that we investigated. Given other evidence of prolonged dysregulation of the circulating B-cell compartment in patients who delay initiation of ART, the inability to return to normalcy and continued immune dysfunction could play a role in HIV-associated mortality and morbidity despite viral suppression and many long term sequela such as persistent immune activation and inflammation with diminished response to vaccines, higher incidence of cardiovascular disease and aging (65).

In LN, we and others have demonstrated that chronic HIV viremia is associated with an accumulation of GCBC and ASC and a depletion of MBC (21, 22, 26, 54, 59). In the current study, where all pre-ART patients were experiencing both chronic HIV viremia and advanced disease, their LN contrasted with other conditions of chronic viremia by an absence of follicular hyperplasia and low frequencies of GCBC, although elevated ASC and reduced IgD^+^ MBC were observed. Following reduction of HIV viremia by ART, the HIV^+^ patients experienced a rapid expansion of both GCBC and DNBC while ASC and MBC remained unchanged. It has been hypothesized that an increase in DNBC correlates with chronic inflammation and thought to represent an extrafollicular albeit T-dependent response (66). However, these studies were performed on PBMC and only recently have LN derived DNBC been investigated, in patients who succumbed to COVID-19 during acute illness (52). In the COVID-19 study, it was notable that the increased frequencies of DNBC were observed in the absence of GC, reduced GCBC and elevated TNF-α. While these observations are consistent with extrafollicular expansion of DNBC, our findings suggest there may be other conditions that favor DNBC expansion. In our HIV^+^ patients, both DNBC and GCBC were expanded post-ART, highly correlated with one another, and occurred as markers of inflammation were decreasing, including TNF-α. Whether these seemingly opposing events occurring simultaneously post-ART, namely LN hyperplasia and decreased inflammation, reflect ongoing immune reconstitution modulated by years of insult to the immune system will require further investigation. There was evidence of some degree of effective B-cell reconstitution post-ART, namely increased expression of IL4R and CD80 on LN B cells, both known to promote prolonged B-T cell interactions essential for efficient GC reactions (53). Yet CD95, a marker of apoptosis, remained elevated.

Transcriptional analyses demonstrated that as immune reconstitution was occurring, there was a concomitant and profound decrease in both inflammatory and IFN responses. IFN signaling was the most differentially regulated pathway following reduction of HIV viremia by ART, reversing the widespread expression of type I IFN and IFN-stimulated genes (ISG) that have been associated with HIV viremia (67, 68). Type I IFN has also been implicated in the immunopathogenesis of advanced HIV disease (69, 70); several studies have indicated that the expression of type I IFN and ISG correlate with higher viral loads, increased immune activation and more rapid disease progression (67, 71–73). Despite the downregulation of pathways associated in HIV-induced activation and inflammation post-ART, several LN abnormalities persisted, including indicators of poor survival of LN Tfh and B cells, and evidence of continued fibrosis, which has been hypothesized to impede recovery of naïve T cells (11).

While changes in BCR diversity and other metrics were limited post-ART, either a reflection of insufficient time or ineffective T-cell help, there was nonetheless evidence of a dynamic process. The negative correlation observed between *IGHV* clonality and frequency of LN GCBC suggested a greater diversity (lower clonality) in the BCR was linked to a greater expansion of GCBC. Furthermore, comparisons between PB and LN identified strong evidence of compartmentalization of BCR repertoires at both timepoints and alterations in immunoglobulin gene usage. Regarding specific *IGHV* families, *IGHV01-69* increased while *IGHV04-34* decreased from pre- to post-ART in LN. The biased use of *IGHV01-69* has been shown to track with protective antibody responses to infections and vaccines (56), thus its increased usage post-ART could help patients mount better antibody responses. However, *IGHV01-69* usage decreased post-ART in PB, an indication there may have been redistribution or involvement of other lymphoid tissues. In contrast, *IGHV04-34* usage decreased in both LN and PB post-ART. This family has been associated with autoimmunity, and by extension, an inability of the immune system to regulate autoreactive B cells (57). Given that numerous immunodeficiencies, including in HIV disease, are often associated with the development of autoimmunity (74, 75), the decrease in *IGHV04-34* usage post-ART in both PB and LN is another indication of that at least some elements of effective immune reconstitution were occurring.

This study has offered a unique window into the early dynamics of immune reconstitution following initiation of ART in advanced HIV disease. The results indicate a mixed picture, with evidence of both effective and ineffective recovery. Whether the latter would reverse given more time is unknown and a limitation of our study. However, given that serial LN biopsies are not feasible in humans, realistic approaches going forward could be to perform similar studies in HIV^+^ patients who begin ART earlier in disease and in other conditions of lymphopenia where immune reconstitution is expected to occur, such as following transplantation in people whose immune system has been fully or partially ablated. A better understanding of the early events of immune reconstitution under different conditions would help elucidate which factors are involved in dictating long term outcomes. Underlying lymphopenia, inflammation, altered homeostasis and comorbidities, some of which may be common across different conditions and others unique to each one may be important determining factors. A complication of our study was the wide spectrum of comorbidities, each low in number and difficult to attribute to any particular effect, and perhaps related to that, the extensive histological findings that likely reflected unavoidable differences between participants in both timing of the biopsy and response rates post-ART. Despite these limitations, one very strong observation emerged, that initiation of ART in advanced HIV disease leads to a rapid expansion in the LN of GCBC, DNBC and Tfh cells. Whether these are the first essential steps to long term effective immunologic recovery will need to be investigated in other conditions of lymphopenia and through further studies in our HIV^+^ cohort, addressing response to vaccination, reduction in co-infections and development of comorbidities.

## Data Availability Statement

The datasets presented in this study can be found in online repositories. The names of the repository/repositories and accession number(s) can be found in the article/**Supplementary Material**.

## Ethics Statement

The studies involving human participants were reviewed and approved by National Institutes of Health IRB. The patients/participants provided their written informed consent to participate in this study.

## Author Contributions

IS (clinical protocol principal investigator) and SM developed the study design, evaluated and interpreted data and helped draft the manuscript. C-SW and CB performed experiments, analyzed and graphed data and drafted the manuscript. SL, LP, FA, ED, SA, KV, CD, and AR performed experiments, analyzed and interpreted data and helped prepare the manuscript. IS, EL, MM, AL, FG, MS, CS, and MA provided clinical care and data acquisition. JD performed surgical lymph node resection. SP and TZ performed the histological analyses and helped prepare the manuscript. All authors reviewed the manuscript. All authors contributed to the article and approved the submitted version.

## Funding

This research was supported by the Intramural Research Program of NIAID, NIH. This project has been funded in whole or in part with federal funds from the National Cancer Institute, National Institutes of Health, under Contract No. 75N91019D00024 (FFRDC contract from 8/31/19 – present). The content of this publication does not necessarily reflect the views or policies of the Department of Health and Human Services, nor does mention of trade names, commercial products, or organizations imply endorsement by the U.S. Government.

## Conflict of Interest

Author AR was employed by company Leidos Biomedical Research Inc.

The remaining authors declare that the research was conducted in the absence of any commercial or financial relationships that could be construed as a potential conflict of interest.

## Publisher’s Note

All claims expressed in this article are solely those of the authors and do not necessarily represent those of their affiliated organizations, or those of the publisher, the editors and the reviewers. Any product that may be evaluated in this article, or claim that may be made by its manufacturer, is not guaranteed or endorsed by the publisher.

## Supplementary Material

Supplementary Figure 1 Longitudinal clinical measurements before and after ART. Participant measurements of **(A)** viral load, **(B)** CD4 T cells, **(C)** CD8 T cells and **(D)** CD19 B cells were determined pre-ART (n = 40) and 6-8 weeks post-ART (n = 30). ****p <* 0.001, *****p <* 0.0001 by bootstrapped Welch Two Sample t-test with 10,000 iterations.

Supplementary Figure 2 CD4 T-cell subsets and representative phenotypic profiles. **(A)** Flow cytometry gating strategy used to identify CD4 T-cell subsets: naïve (CD27^+^CD45RO^-^), central memory (CD27^+^CD45RO^+^), effector memory (CD27^-^CD45RO^+^), effector (CD27^-^CD45RO^-^), or Treg (CD25^+^ FOXP3^+^) in PBMC of HIV^+^ participants. **(B-D)** Representative **(B)** LNMC Tfh and **(C) P**BMC cTfh cells (CXCR5^+^PD-1^hi^) among CD3^+^CD4^+^CD45RO^+^ T cells for HC and HIV^+^ pre- and post-ART participants. **(D)** PBMC cTfh subsets: Tfh1(CXCR3^+^CCR6^−^ cells), Tfh2 (CXCR3^−^CCR6^−^ cells), Tfh17 (CXCR3^−^CCR6^+^), and Tfh1/17 (CXCR3^+^CCR6^+^) and frequencies of HC (n = 6) post-ART cTfhs (n = 6). **p <* 0.05, ***p <* 0.01 by Mann-Whitney U test.

Supplementary Figure 3 Effect of IRIS on CD4 T cells. **(A, B)** Frequencies of CD4 T-cell subsets (naïve, effector, central memory, effector memory, Treg and Tfh) in LNMC of HIV^+^ participants with and without IRIS **(A)** pre- (n = 12 with IRIS; n = 17 without IRIS for CD4 subsets) and with pre- (n = 12 with IRIS; n =24 without IRIS for Tfhs) **(B)** post-ART (n = 11 with IRIS; n = 15 without IRIS for CD4 subsets) and with post- (n = 17 with IRIS; n =10 without IRIS for Tfhs) were determined using flow cytometry. Bootstrapped Welch Two Sample t-test with 10,000 iterations was performed for comparisons; data are not statistically significant unless noted.

Supplementary Figure 4
*In vitro* analysis of cTfh cells. CXCR5^+^CD45RO^+^CD4^+^ T cells from HC (n = 5) or post-ART HIV^+^ (n = 5) participants were cocultured with unrelated HC CD19^+^ B cells. **(A)** Flow cytometry gating strategy used to identify B cells subsets: naïve B cells (CD19^+^IgD^+^CD27^-^), memory B cells (CD19^+^IgD^-^CD27^+^), and plasmablasts or plasma cells (CD19^+^CD38^+^CD27^+^). **(B)** *In vitro* analysis of non-cTfh cells. CXCR5^-^CD45RO^+^CD4^+^ T cells from HC (n = 5) or post-ART HIV^+^ (n = 5) participants were cocultured with unrelated HC CD19^+^ B cells. After 7 days of coculture, the B cells were phenotyped for differentiation status: naïve (IgD^+^CD27^-^), memory (IgD^-^CD27^+^), antibody-secreting cells (ASC; CD27^+^CD38^hi^) and percent change was calculated from wells containing B cells alone. **(C)** Absolute numbers of cTfh cells in culture on day 7. Mann-Whitney U test was performed for comparisons. **(D)** *In vitro* analysis of cTfh cells. CXCR5^+^CD45RO^+^CD4^+^ T cells from HC (n = 3) or post-ART HIV^+^ (n = 3) participants were cocultured with unrelated HC CD19^+^ B cells for 3 days. CD4^+^ T were assayed for Annexin V and Ki-67. **(E)** ex vivo frequency of Ki-67 in pre-ART (n = 15), post-ART (n = 15) and HC (n = 6) of cTfh cells (CXCR5^+^CD45RO^+^CD4^+^). Bootstrapped Welch Two Sample t-test with 10,000 iterations was performed for comparisons; data are not statistically significant unless noted.

Supplementary Figure 5 B-cell subsets and percent area occupied by CD20^+^ cells in the LN. **(A)** Gating strategy used to identify PBMC B-cell subsets: antibody-secreting cells (ASC; CD21^-^CD27^hi^), activated memory (CD21^lo^CD27^+^), resting memory (CD21^+^CD27^+^), tissue-like memory (CD21^lo^CD27^-^), naïve (CD21^+^CD27^-^), and immature/transitional (CD21^lo/hi^CD10^+^) B cells. **(B)** Gating strategy used to identify LNMC B-cell subsets: antibody-secreting cells (ASC; IgD^-^CD38^hi^) and germinal center (GCBC; IgD^-^CD38^+^), IgD^-^ memory (IgD^-^CD27^+^), naïve (IgD^+^CD27^-^), IgD^+^ memory (IgD^+^CD27^+^), and IgD^-^CD27^-^ double negative (DNBC) B cells. **(C, D)** Quantitative image analysis of the percentage of area of the B-cell follicle (BCF) **(C)** and T-cell zone (TCZ) **(D)** that stained positive for CD20 (n = 8 pre- and post-ART pairs); IRIS patients indicated by circles. Wilcoxon signed rank test was performed.

Supplementary Figure 6 Effect IRIS on B cells. **(A, B)** Frequencies of B-cell subsets (naïve, IgD^-^ memory, IgD^+^ memory, germinal center (GCBC), and antibody-secreting cell (ASC) in LNMC of HIV^+^ participants with and without IRIS **(A)** pre- (n = 14 with IRIS; n = 22 without IRIS) and **(B)** post-ART (n = 9 with IRIS; n = 19 without IRIS) were determined using flow cytometry. Bootstrapped Welch Two Sample t-test with 10,000 iterations was performed for comparisons; data are not statistically significant unless noted.

Supplementary Figure 7 Biomarker evaluation and correlations with LNMC. **(A)** Comparison of concentrations of plasma cytokines, chemokines and soluble factors of HIV^+^ participants pre- and post-ART (n = 8 or 17). **p <* 0.05, ***p <* 0.01 by Wilcoxon signed rank test; ns, not significant. **(B)** Correlation matrix depicting the Tfh, GCBC and IgD^-^CD27^-^ (DNBC) cells in LNMC and concentrations of cytokines, chemokines and soluble factors in the plasma of HIV^+^ participants pre- and post-ART. **p* < 0.05, ***p* < 0.01, ****p* < 0.001 by Spearman’s rank correlation; data are not statistically significant unless noted.

Supplementary Figure 8 Additional BCR analyses. **(A)** Total (left) and normalized (right) number of productive unique B-cell receptor (BCR) sequences determined for peripheral blood (PB) and lymph node (LN) samples of nine HIV^+^ pre- and post-ART participants. **(B)** Simpson clonality determined for PB and LN samples of nine HIV^+^ pre- and post-ART participants. **(C)** Heatmap of Morisita index for one representative donor. **(D)** Representative scatterplots of Ig heavy chain rearrangements in the PB and LN. **(E)** Frequency of IGVH gene families *IGHV04*, *IGHV05*, *IGHV06*, and *IGHV07* in the PB and LN of nine HIV^+^ pre- and post-ART participants. **(F)** Antibody titers for influenza and VZV in HIV^+^ sera from week 0, 4, and 24 (n = 24). **p <* 0.05 by Wilcoxon signed rank test; data are not statistically significant unless noted.
