# Supplementary material for: Rapid Emergence of T Follicular Helper and Germinal Center B Cells Following Antiretroviral Therapy in Advanced HIV Disease
Source: Front Immunol. 2021 Dec 1;12:752782. doi: 10.3389/fimmu.2021.752782 (PMC8686113; doi:10.3389/fimmu.2021.752782)
Supplement: Supplementary Figure 1 — Longitudinal clinical measurements before and after ART. Participant measurements of (A) viral load, (B) CD4 T cells, (C) CD8 T cells and (D) CD19 B cells were determined pre-ART (n = 40) and 6-8 weeks post-ART (n = 30). ***p < 0.001, ****p < 0.0001 by bootstrapped Welch Two Sample t-test with 10,000 iterations. [file Presentation_1.pptx]

## Slide 1
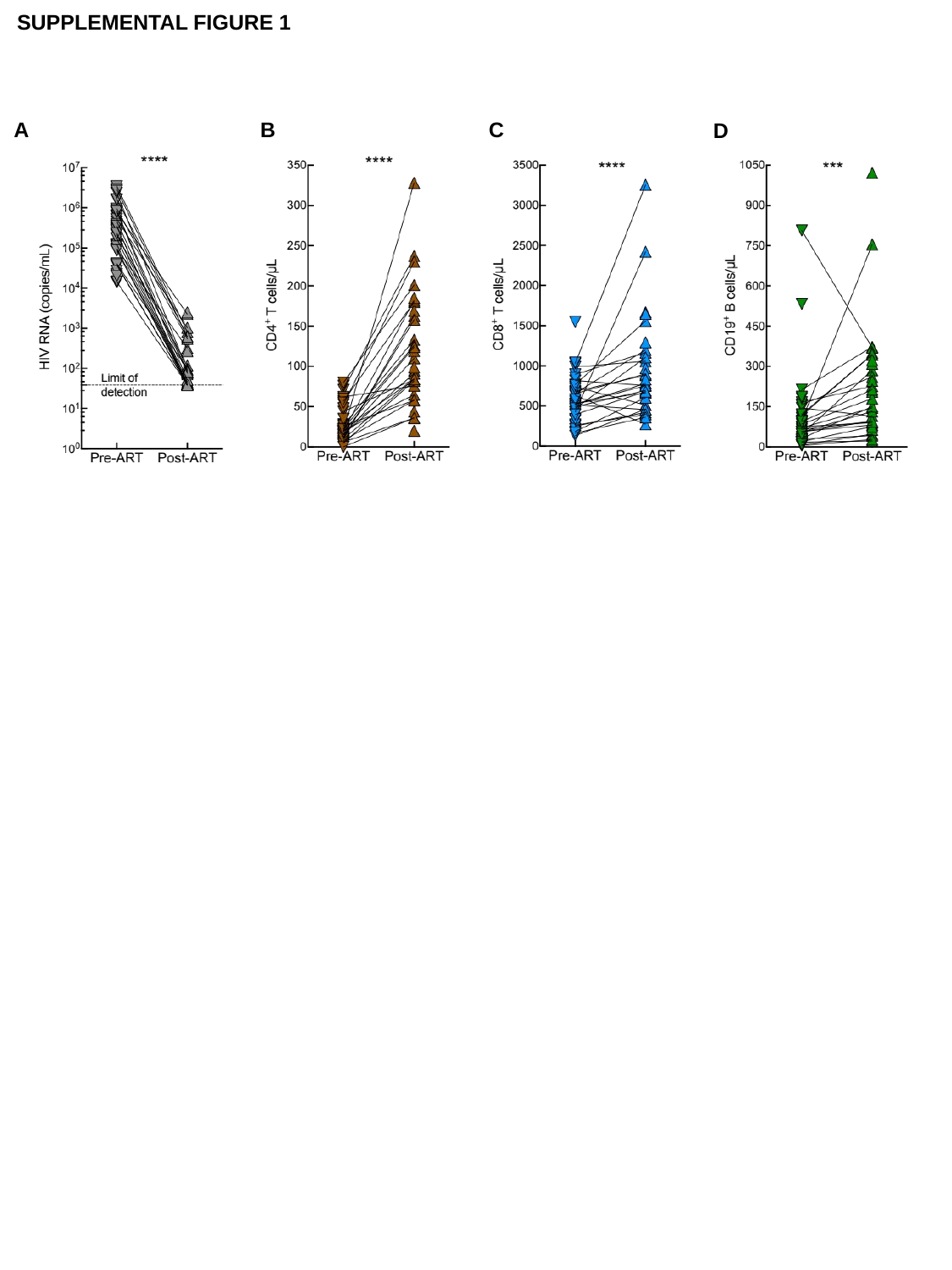

SUPPLEMENTAL FIGURE 1
C
A
B
D

## Slide 2
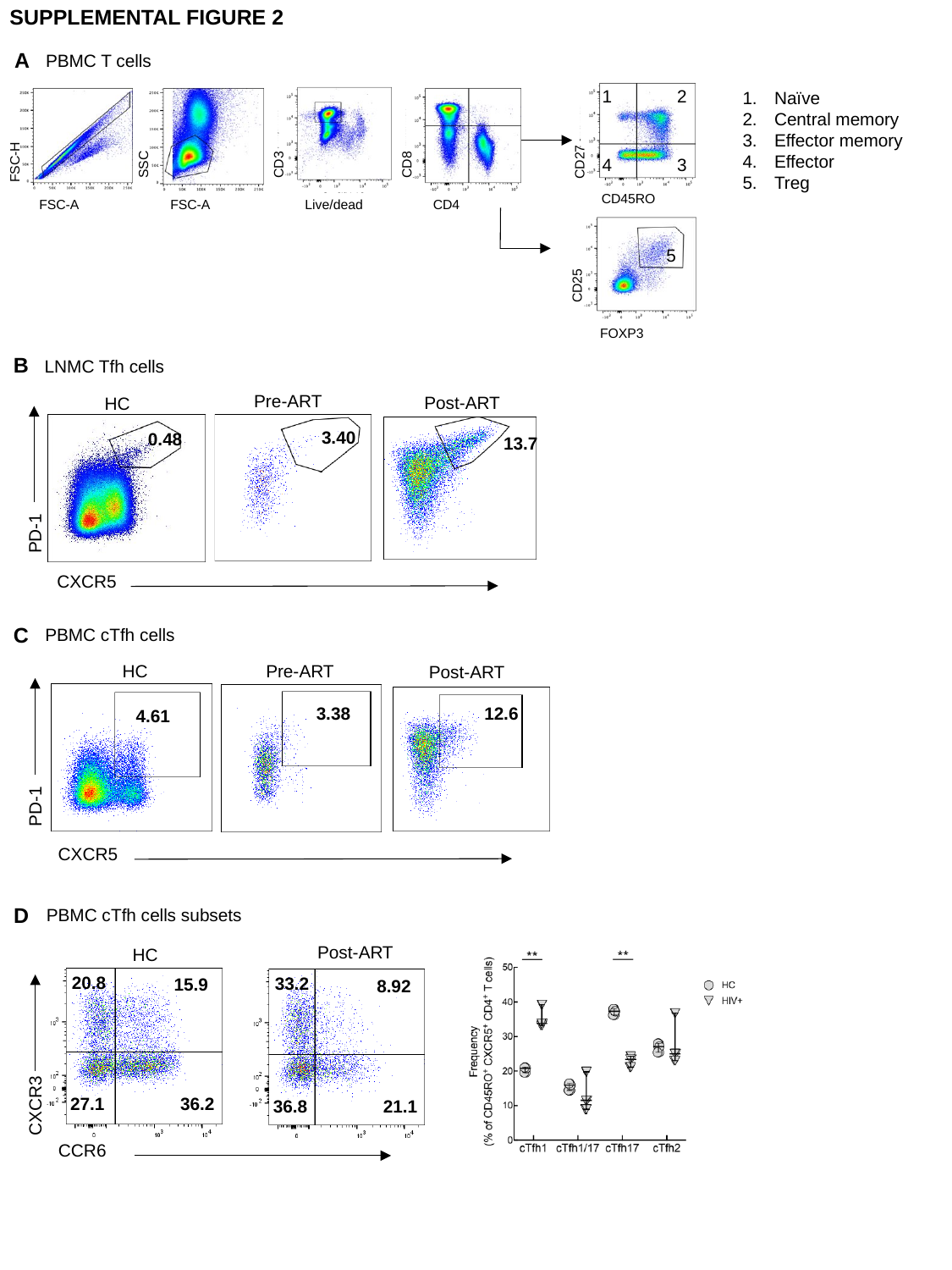

SUPPLEMENTAL FIGURE 2
A
PBMC T cells
1
2
Naïve
Central memory
Effector memory
Effector
Treg
SSC
CD3
CD8
CD27
FSC-H
4
3
CD45RO
FSC-A
FSC-A
Live/dead
CD4
5
CD25
FOXP3
B
LNMC Tfh cells
Pre-ART
HC
0.48
PD-1
CXCR5
Post-ART
3.40
13.7
C
PBMC cTfh cells
HC
Pre-ART
Post-ART
3.38
12.6
4.61
PD-1
CXCR5
D
PBMC cTfh cells subsets
Post-ART
HC
20.8
33.2
15.9
8.92
HC
CXCR3
27.1
36.2
36.8
21.1
CCR6

## Slide 3
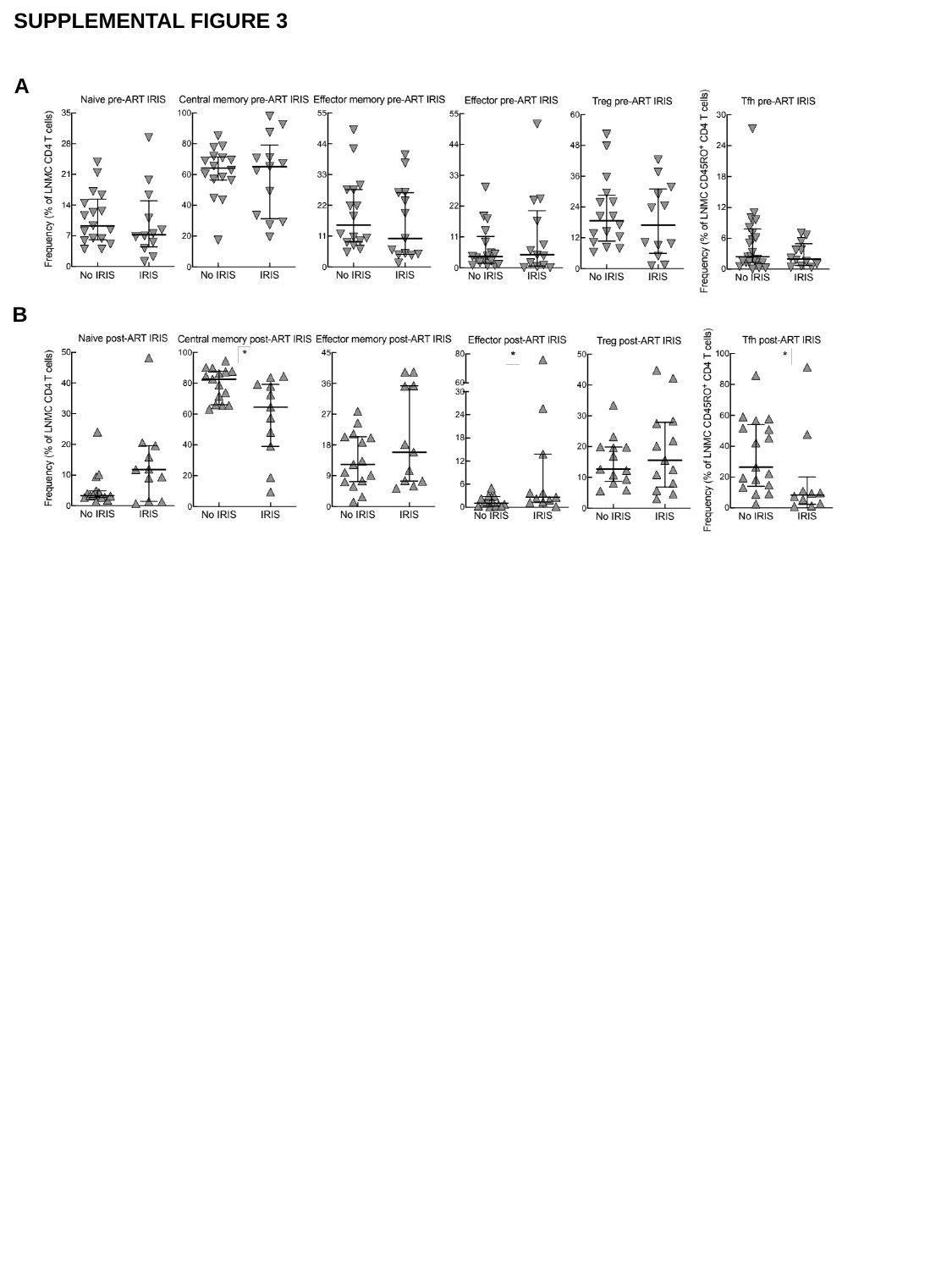

SUPPLEMENTAL FIGURE 3
A
B

## Slide 4
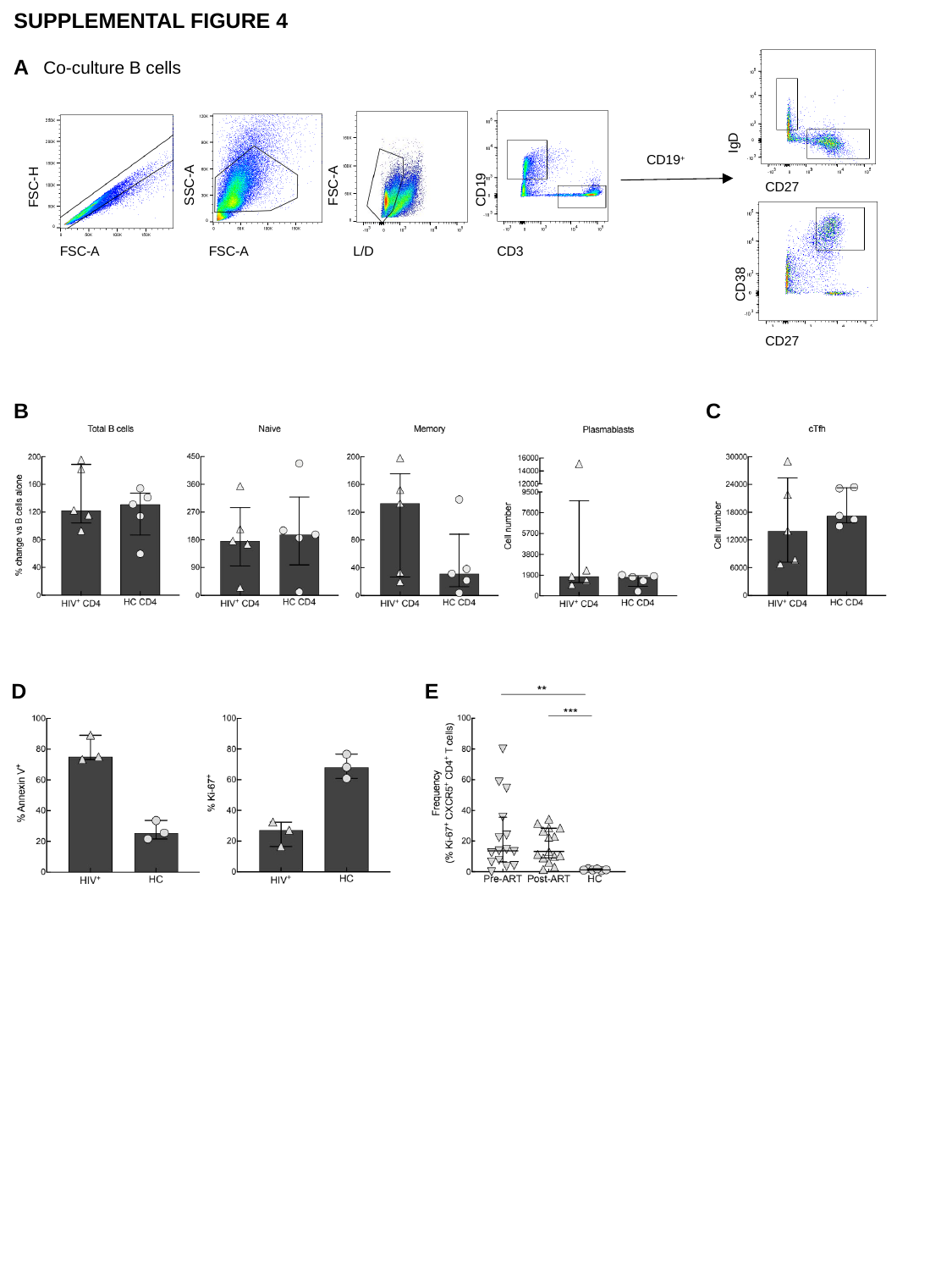

SUPPLEMENTAL FIGURE 4
A
IgD
CD19+
SSC-A
FSC-A
FSC-H
CD19
CD27
FSC-A
CD3
FSC-A
L/D
CD38
CD27
Co-culture B cells
B
C
D
E

## Slide 5
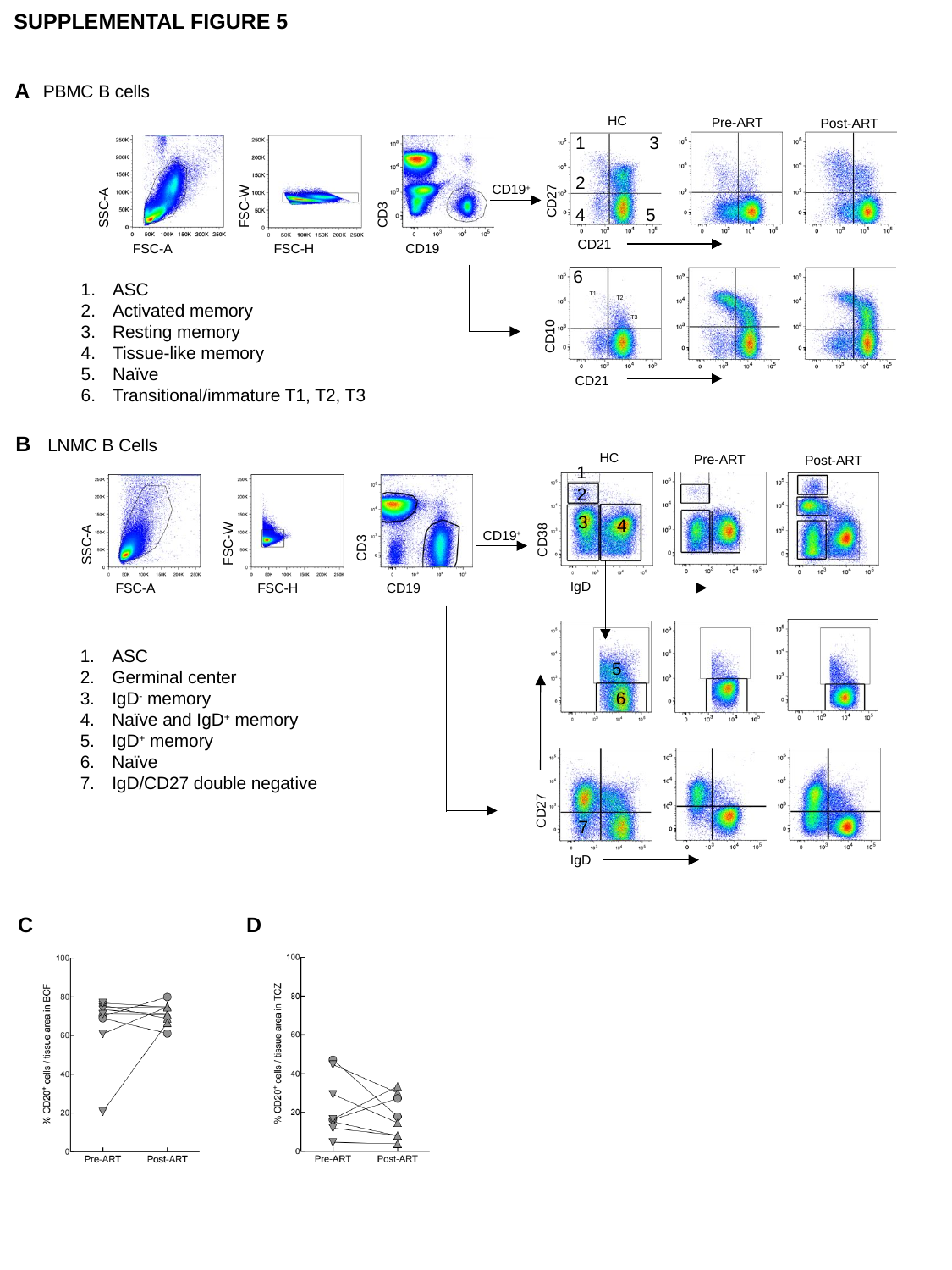

SUPPLEMENTAL FIGURE 5
A
PBMC B cells
HC
Pre-ART
Post-ART
1
3
2
CD19+
CD27
SSC-A
FSC-W
CD3
4
5
CD21
FSC-A
FSC-H
CD19
6
T1
T2
T3
ASC
Activated memory
Resting memory
Tissue-like memory
Naïve
Transitional/immature T1, T2, T3
CD10
CD21
B
LNMC B Cells
HC
Pre-ART
Post-ART
1
2
3
4
CD38
CD19+
CD3
SSC-A
FSC-W
IgD
FSC-A
FSC-H
CD19
ASC
Germinal center
IgD- memory
Naïve and IgD+ memory
IgD+ memory
Naïve
IgD/CD27 double negative
5
6
CD27
7
IgD
D
C

## Slide 6
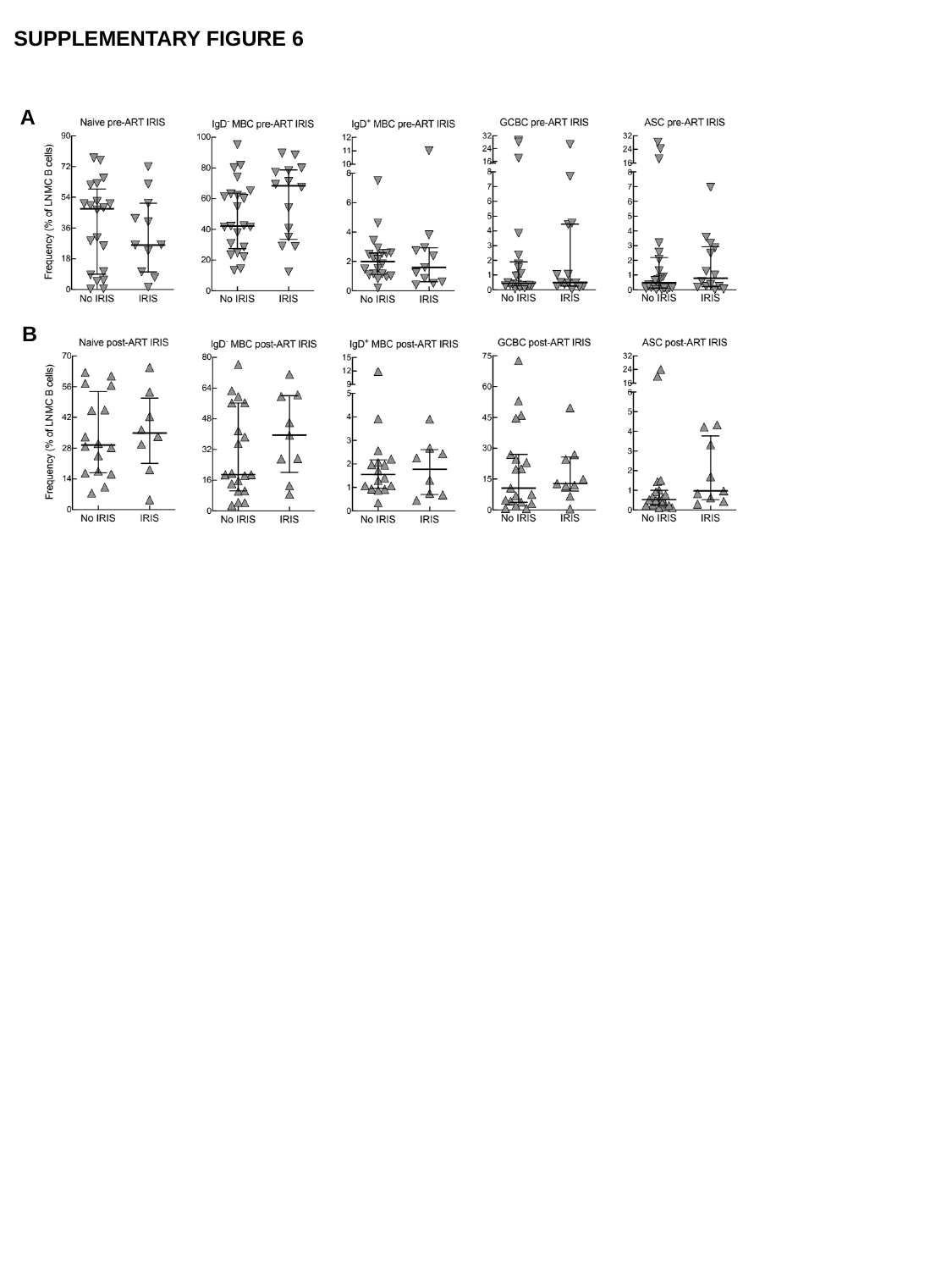

SUPPLEMENTARY FIGURE 6
A
B

## Slide 7
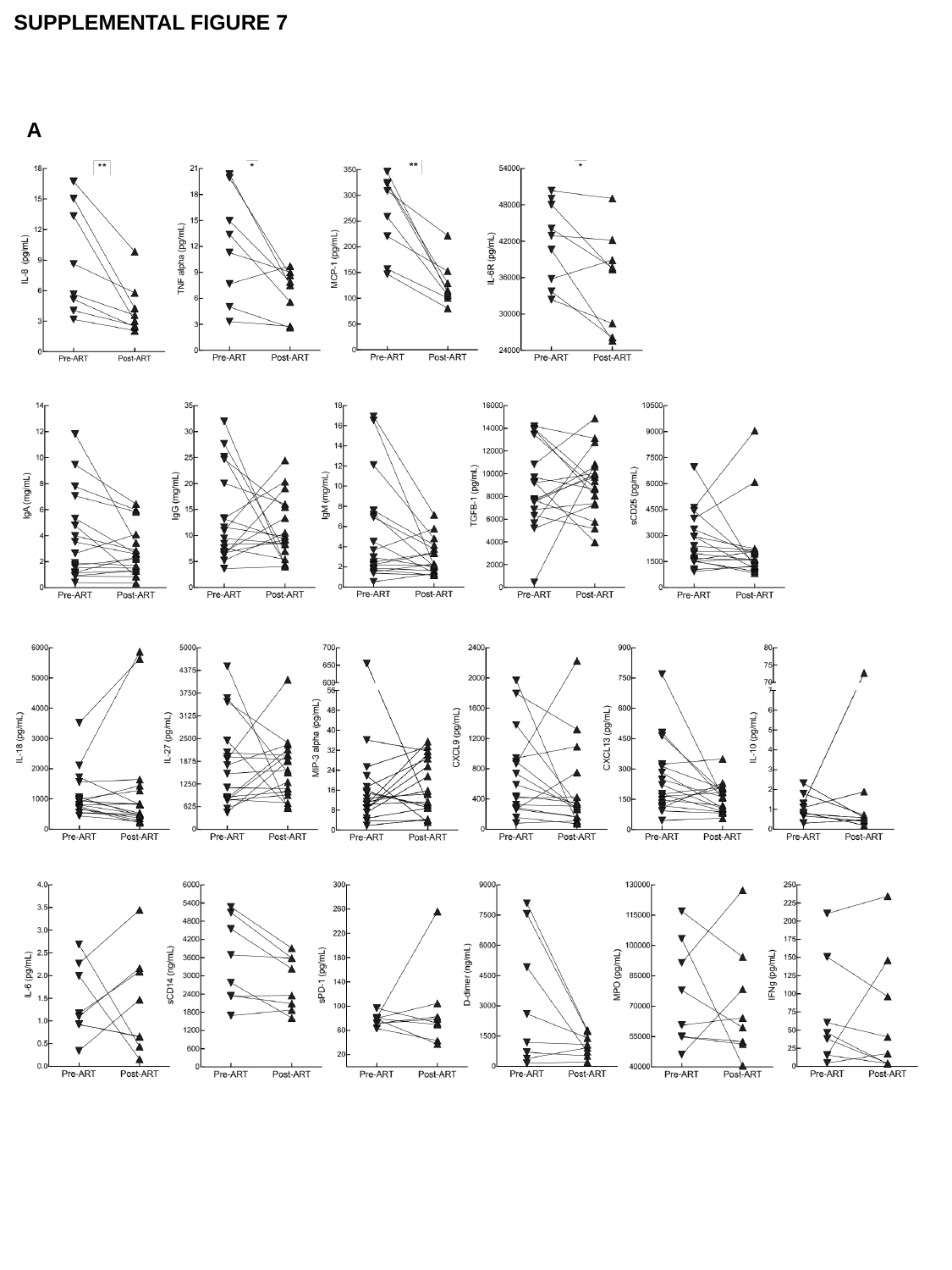

SUPPLEMENTAL FIGURE 7
A

## Slide 8
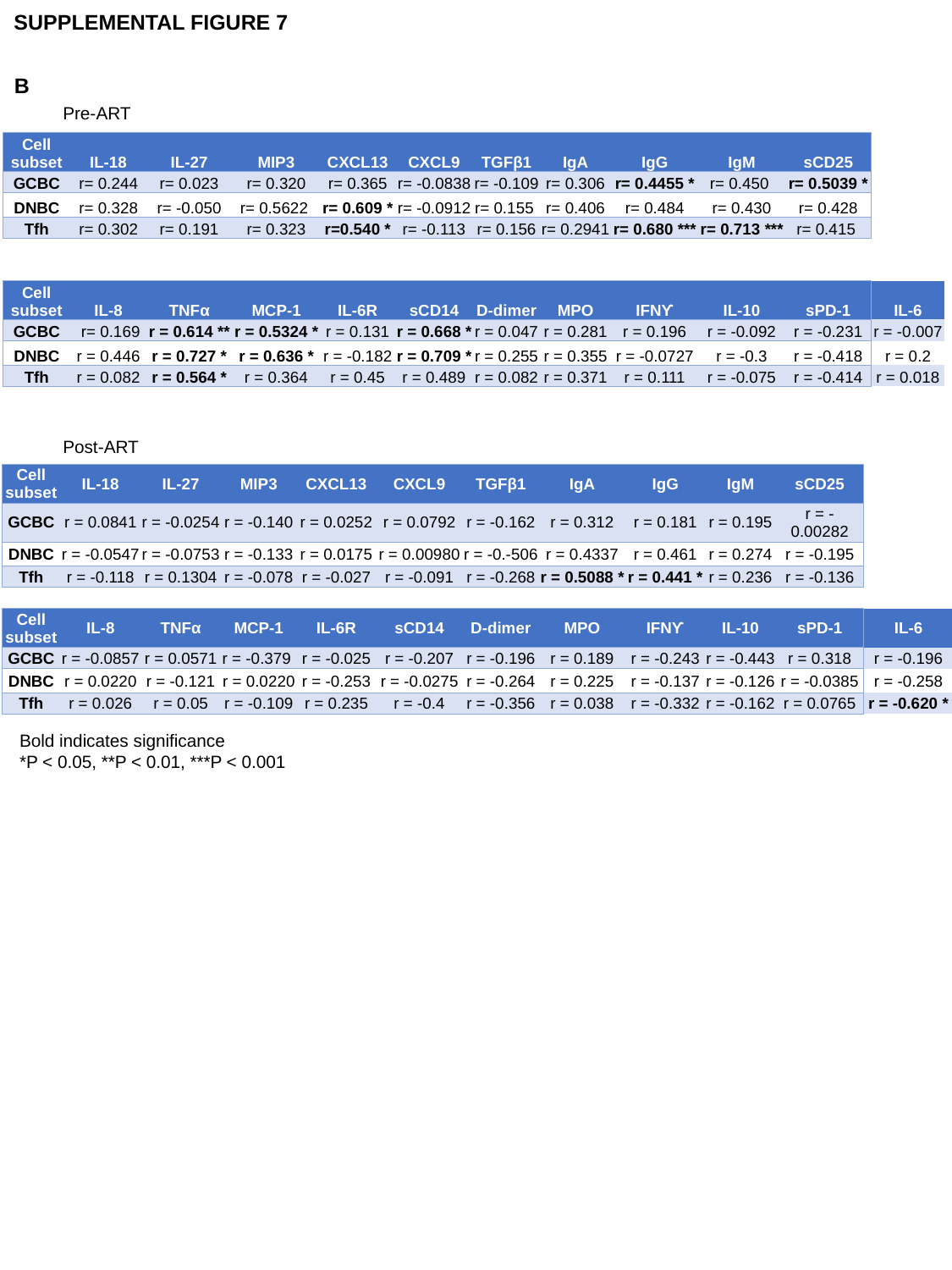

SUPPLEMENTAL FIGURE 7
B
Pre-ART
| Cell subset | IL-18 | IL-27 | MIP3 | CXCL13 | CXCL9 | TGFβ1 | IgA | IgG | IgM | sCD25 | |
| --- | --- | --- | --- | --- | --- | --- | --- | --- | --- | --- | --- |
| GCBC | r= 0.244 | r= 0.023 | r= 0.320 | r= 0.365 | r= -0.0838 | r= -0.109 | r= 0.306 | r= 0.4455 \* | r= 0.450 | r= 0.5039 \* | |
| DNBC | r= 0.328 | r= -0.050 | r= 0.5622 | r= 0.609 \* | r= -0.0912 | r= 0.155 | r= 0.406 | r= 0.484 | r= 0.430 | r= 0.428 | |
| Tfh | r= 0.302 | r= 0.191 | r= 0.323 | r=0.540 \* | r= -0.113 | r= 0.156 | r= 0.2941 | r= 0.680 \*\*\* | r= 0.713 \*\*\* | r= 0.415 | |
| | | | | | | | | | | | |
| | | | | | | | | | | | |
| Cell subset | IL-8 | TNFα | MCP-1 | IL-6R | sCD14 | D-dimer | MPO | IFNϒ | IL-10 | sPD-1 | IL-6 |
| GCBC | r= 0.169 | r = 0.614 \*\* | r = 0.5324 \* | r = 0.131 | r = 0.668 \* | r = 0.047 | r = 0.281 | r = 0.196 | r = -0.092 | r = -0.231 | r = -0.007 |
| DNBC | r = 0.446 | r = 0.727 \* | r = 0.636 \* | r = -0.182 | r = 0.709 \* | r = 0.255 | r = 0.355 | r = -0.0727 | r = -0.3 | r = -0.418 | r = 0.2 |
| Tfh | r = 0.082 | r = 0.564 \* | r = 0.364 | r = 0.45 | r = 0.489 | r = 0.082 | r = 0.371 | r = 0.111 | r = -0.075 | r = -0.414 | r = 0.018 |
Post-ART
| Cell subset | IL-18 | IL-27 | MIP3 | CXCL13 | CXCL9 | TGFβ1 | IgA | IgG | IgM | sCD25 | |
| --- | --- | --- | --- | --- | --- | --- | --- | --- | --- | --- | --- |
| GCBC | r = 0.0841 | r = -0.0254 | r = -0.140 | r = 0.0252 | r = 0.0792 | r = -0.162 | r = 0.312 | r = 0.181 | r = 0.195 | r = -0.00282 | |
| DNBC | r = -0.0547 | r = -0.0753 | r = -0.133 | r = 0.0175 | r = 0.00980 | r = -0.-506 | r = 0.4337 | r = 0.461 | r = 0.274 | r = -0.195 | |
| Tfh | r = -0.118 | r = 0.1304 | r = -0.078 | r = -0.027 | r = -0.091 | r = -0.268 | r = 0.5088 \* | r = 0.441 \* | r = 0.236 | r = -0.136 | |
| | | | | | | | | | | | |
| Cell subset | IL-8 | TNFα | MCP-1 | IL-6R | sCD14 | D-dimer | MPO | IFNϒ | IL-10 | sPD-1 | IL-6 |
| GCBC | r = -0.0857 | r = 0.0571 | r = -0.379 | r = -0.025 | r = -0.207 | r = -0.196 | r = 0.189 | r = -0.243 | r = -0.443 | r = 0.318 | r = -0.196 |
| DNBC | r = 0.0220 | r = -0.121 | r = 0.0220 | r = -0.253 | r = -0.0275 | r = -0.264 | r = 0.225 | r = -0.137 | r = -0.126 | r = -0.0385 | r = -0.258 |
| Tfh | r = 0.026 | r = 0.05 | r = -0.109 | r = 0.235 | r = -0.4 | r = -0.356 | r = 0.038 | r = -0.332 | r = -0.162 | r = 0.0765 | r = -0.620 \* |
Bold indicates significance
*P < 0.05, **P < 0.01, ***P < 0.001

## Slide 9
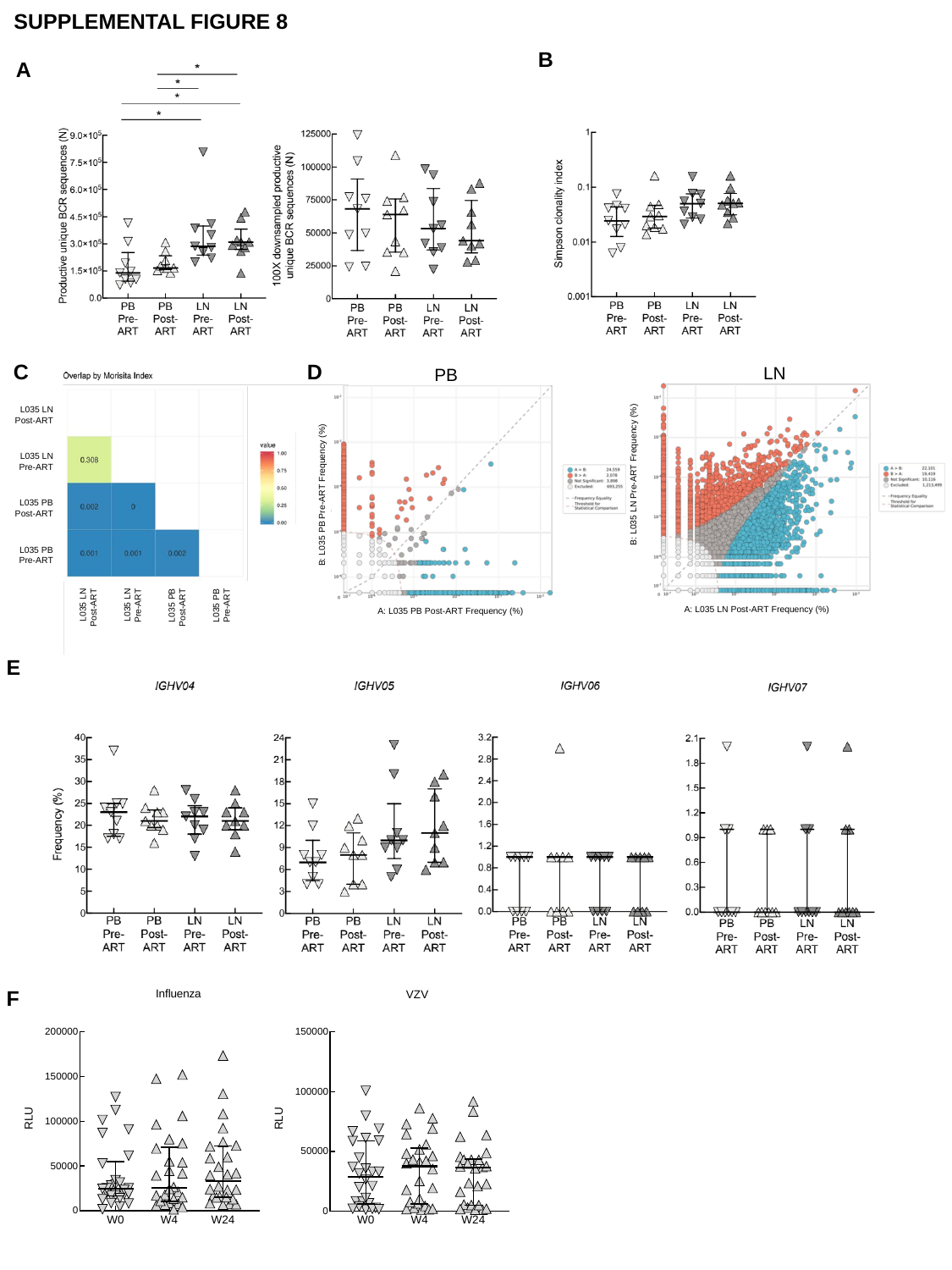

SUPPLEMENTAL FIGURE 8
B
A
D
C
LN
PB
L035 LN Post-ART
L035 LN Pre-ART
L035 PB Post-ART
L035 PB Pre-ART
L035 PB Pre-ART
L035 PB Post-ART
L035 LN Post-ART
L035 LN Pre-ART
B: L035 PB Pre-ART Frequency (%)
A: L035 PB Post-ART Frequency (%)
B: L035 LN Pre-ART Frequency (%)
A: L035 LN Post-ART Frequency (%)
E
F
